# Newborns' Asymmetrical Processing of Order From Sequentially Presented Magnitudes

**DOI:** 10.1111/cdev.70025

**Published:** 2025-08-06

**Authors:** Martina Arioli, Valentina Silvestri, Angelo Petrelli, Daniela Morniroli, Maria Lorella Giannì, Hermann Bulf, Viola Macchi Cassia

**Affiliations:** ^1^ Department of Psychology Università Degli Studi di Milano‐Bicocca Milan Italy; ^2^ Department of Clinical Sciences and Community Health University of Milano Milan Italy; ^3^ Neonatal Intensive Care Unit Fondazione IRCCS Cà Granda Ospedale Maggiore Policlinico Milan Italy

**Keywords:** human newborns, increasing, magnitudes, ordinal computation, visual sequences

## Abstract

Four‐month‐old infants extract ordinal information in number‐based and size‐based visual sequences, provided that magnitude changes involve increasing relations. Here the ontogenetic origins of ordinal processing were investigated between 2018 and 2022 by testing newborns' discrimination of reversal in numerosity (Experiment 1, *N* = 22 White, 11 females), numerical order in the presence of redundant non‐numerical quantitative cues (Experiment 2, *N* = 44 White, 23 females), or size‐based order (Experiment 3, *N* = 44 White, 21 females). Newborns' post‐habituation preferences revealed successful discrimination only when both numerical (items' number) and non‐numerical (items' size) cues concurrently changed, and following habituation to increasing order (*p* = 0.017, *η* ^2^_p_ = 0.135). These findings, along with evidence from older infants and non‐human animals, suggest continuity in magnitude representation across ontogenetic and phylogenetic levels.

## 1 Introduction

During the last few decades, developmental science has provided strong evidence that the ability to encode and represent quantitative attributes of the environment and to operate over such representations emerges very early in human ontogeny. The interest in understanding the developmental roots of these abilities in early infancy has taken place in the context of modern cognitive science that considers the dimensions of number, space, and time as critical environmental attributes used by nonhuman and human animals to adapt to and represent the world (e.g., Carey 2011).

Animals of many species (e.g., Cantlon 2012; Gatto et al. 2022; Rugani et al. 2020) and human infants in their first year of life have been shown to discriminate between the numerosities of visual–spatial arrays or auditory–temporal sequences, between the sizes of visual objects or between the temporal durations of acoustic, visual, or audiovisual events (see review by Feigenson 2011). In preverbal infants, these abilities have been studied above all using the habituation/dishabituation and familiarization procedures based on the recording of looking time measures (see review by Colombo and Mitchell 2009). Such procedures allow measuring both perceptual learning and recognition memory, which both reveal that a form of mental representation of the stimuli/event presented is being stored in memory (Pascalis and de Haan 2014). For instance, 6‐month‐old infants who have been habituated to multiple arrays of 8 objects and are then tested with a new array of 8 novel objects in some trials or 4 novel objects in other trials show increased attention to the trials containing the novel object numerosity (e.g., Xu and Spelke 2000). Although the contribution of cortical and subcortical networks to infants' perception of numerosity is currently debated (Blumberg and Adolph 2023), this type of evidence leads researchers to infer that infants have extracted the approximate cardinal value of the familiarized stimuli across variations in their perceptual appearance (e.g., shape, size, color) and continuous quantitative features (e.g., physical size, contour length). Also, they stored in working memory a representation of the numerical value that is unbounded by the limits of immediate perception, to which new instances of the same numerical value can be associated.

Further evidence of the abstract nature of preverbal representations of number comes from the observation that such representation supports intermodal comparison, allowing infants to compare numerosities experienced in one sensory modality (e.g., audition) to numerosities experienced in another (e.g., vision) (Feigenson 2011). Six‐month‐old infants familiarized with a one‐to‐one pairing of 6 objects and sounds exhibited increased attention to number mismatch trials, where the auditory and visual numerosities did not correspond, during subsequent testing with 8 sounds and 8 or 4 objects. Critically, infants in this study were forced to use memory as they heard and saw numerical stimuli at different times. This allowed researchers to conclude that infants can form and maintain in working memory genuinely abstract representations with approximate numerical content, just like older children (e.g., Barth et al. 2006) and adults (e.g., Barth et al. 2003).

The ability to use memory to compare numerical representations is key to computation, as when adding an approximate number of food items to a set of previously perceived items, or when ordering the number of perceptually available items with respect to the number of those previously experienced. Accordingly, preverbal infants (and non‐human animals; e.g., Garland and Low 2014; Livingstone et al. 2014) can operate over representations of both small and large numerosities by adding, subtracting (e.g., Kobayashi et al. 2004; McCrink and Wynn 2004; Wynn 1992) and ordering (e.g., de Hevia et al. 2017; Picozzi et al. 2010) them. For instance, 5‐month‐olds can compute the addition of 1 object and 2 sounds and compare the result with a third array displaying 3 objects or 2 objects, preferring the unexpected, incorrect outcome over the expected, correct one (Kobayashi et al. 2004).

From about the same age, infants have been shown to discriminate ordinal relations (increasing or decreasing) within sets of non‐symbolic numbers when the ratio of numerical changes is sufficiently large. In two studies using different numerosities, Macchi Cassia and colleagues (de Hevia et al. 2017) habituated 4‐month‐old infants to increasing sequences of large numerosities differing one from the other according to a 1:3 ratio. At test, infants generalized habituation responses to new numerical displays arranged in the familiar order while dishabituated to the same displays arranged in a decreasing order, even when non‐numerical continuous variables were controlled for. Similar results were obtained in older infants (i.e., 11 months; Brannon 2002; Suanda et al. 2008).

In these studies, numerical order was operationalized as the progressions (either incrementing or decrementing) between at least three numerosities. Magnitude changes had to repeat at least twice and be consistent within a given sequence (e.g., 6‐18‐54 or 54‐18‐6), so that ordinality emerged from iterative cardinality comparisons between constituent pairs of numerosities. This computation differs from the one taking place in simple numerical comparison and discrimination where infants can succeed by simply perceiving the numerical dissimilarity between two sets of objects (e.g., 8 vs. 16) without needing to compute the ‘greater than’ or ‘less than’ relations between them. Also, non‐symbolic numerosities were sequentially deployed one after the other within each sequence. This required infants to rely on working memory to compute increasing and decreasing numerosity changes as the difference between the numerosity of the perceptually available display and the represented numerosity corresponding to the previously presented display.

Evidence from this work has challenged the view, inspired by Piaget's theory (1954), that the ability to compare and discriminate numerosities precedes ordinal understanding. Within this view, the ability to grasp numerical order would emerge in preschool years through the observation of transformations in the environment, such as adding and removing objects, and the quantitative changes that derive from those transformations (e.g., Cooper Jr 1984; Mix et al. 2002). Infant research has also challenged the idea that infants understand non‐numerical order before grasping numerical order (Brannon 2002; Suanda et al. 2008). While studies showed that 4‐month‐old infants can detect order in non‐symbolic numbers (de Hevia et al. 2017), they also found this ability generalizes equally to physical size (i.e., sequences of shapes varying in surface area; Macchi Cassia et al. 2012), suggesting a common development of ordinal representation for the two quantitative dimensions of size and number.

Despite their ability to perform ordering computations, unlike older infants aged 7–9 months (Macchi Cassia et al. 2017; de Hevia and Spelke 2010; Picozzi et al. 2010), 4‐month‐old infants exhibit an asymmetry in their sensitivity to magnitude changes. Specifically, they succeed at detecting and representing numerical increase but fail with numerical decrease. This is evident in their successful discrimination of a reversal in ordinal direction following habituation to increasing, but not decreasing, numerical sequences (de Hevia et al. 2017). Notably, this asymmetry signature (i.e., success for increasing order and failure for decreasing order) extends to size ordering (Macchi Cassia et al. 2012; but not temporal duration, see de Hevia et al. 2020) as 4‐month‐olds show the same pattern of success with increasing size‐based sequences and failure with decreasing ones.

It has been suggested (see discussion in de Hevia et al. 2017; Macchi Cassia et al. 2012) that this ordinal asymmetry might be a developmental precursor of the ‘addition advantage’, a phenomenon characterized by better performance and earlier acquisition of addition relative to subtraction arithmetic operations. This advantage persists from childhood to adulthood, encompassing both symbolic (e.g., Kamii et al. 2001) and non‐symbolic (e.g., Shinskey et al. 2009) arithmetic performance. With large non‐symbolic sets, adults and young children are more accurate with addition than with subtraction problems (Barth et al. 2006, 2008; Shinskey et al. 2009), and this advantage is shared across cultures (Campbell and Xue 2001). Also, in formal education, addition is considered the foundation for subtraction, and the processing of counting‐down for subtraction is commonly more time‐consuming than the processing of counting‐up for addition (Baroody 1984; Canobi 2005; Fuson 1984).

The link between infants' ordinal asymmetry and the later‐developing addition advantage would not be the only instance of ontogenetic continuity in ordinal representation. For example, the Operational Momentum (OM) phenomenon, by which participants systematically overestimate the outcomes of addition problems and underestimate those of subtraction problems, has been reported during symbolic and non‐symbolic arithmetic in adults (Knops, Viarouge, & Dehaene, 2009; McCrink, Dehaene, & Dehaene‐Lambertz, 2007) and preverbal infants (McCrink and Wynn 2009). In particular, recent evidence has shown that the OM effect in preschoolers (Dunn et al. 2019) and preverbal infants (Macchi Cassia et al. 2016, 2017) extends to ordering operations over magnitudes as well. After habituation to either increasing or decreasing sequences in which number covaried with envelope and cumulative area, 4‐month‐old infants looked longer to sequences that exhibited the familiar order instantiated by novel magnitudes violating the OM generated during habituation—i.e. larger magnitudes for infants habituated to decreasing order, and smaller magnitudes for infants habituated to increasing order (Macchi Cassia et al. 2017).

Despite the asymmetry feature in ordinal processing is not anymore apparent in habituation/dishabituation tasks by the time human infants are aged 7–9 months (de Hevia and Spelke 2010; Picozzi et al. 2010), it has been hypothesized that it might constitute a core processing constraint of the Analog Number System (ANS; Dehaene 2011). The ANS is the cognitive mechanism underlying humans' and animals' ability to represent and operate in an approximate way over large numerosities (≥ 4). As such, ordinal asymmetry may permeate cognitive tasks that rely on such system. Indeed, a well‐documented ‘ascending order advantage’ is evident in adults' performance in comparison tasks. Adults perform better when the smaller quantity is temporally followed by the larger one (increasing order), compared to when the smaller is temporally preceded by the larger one (decreasing order) (Ben‐Meir et al. 2012; Müller and Schwarz 2008). Moreover, the asymmetry signature in ordinal processing expands the list of known cognitive constraints shared by humans and non‐human animals in their processing of numerosities (Cantlon 2012). There is evidence that monkeys, similar to humans, can generalize a previously learned increasing order to new numbers, but they fail this generalization for decreasing order (Brannon, Cantlon, and Terrace 2006a; Brannon, Lutz, and Cordes 2006b; Brannon and Terrace 1998; Cantlon and Brannon 2006; Judge et al. 2005).

The pervasiveness of the asymmetry signature across developmental stages and animal species suggests ontogenetic and phylogenetic continuity (see, e.g., Adachi 2014; Drucker and Brannon 2014; Rugani et al. 2015 for similar evidence of phylogenetic continuity in number‐space mapping). However, a critical gap exists in our understanding of this phenomenon in human infants, as no research has yet explored numerical order processing before 4 months. This leaves the ontogenetic origins of the increasing order advantage unclear.

Researchers who identified such advantage in 4‐month‐old infants hypothesized a link to the well‐documented bias in humans and non‐human animals to prioritize from very early in life visual information specifying ‘looming’ (expanding) over ‘zooming’ (contracting) stimuli (e.g., Van der Weel and van der Meer 2009). This bias refers to the tendency to pay more attention to objects perceived as progressively approaching compared to those receding from the observer. Classic studies have shown that infants exhibit specific behavioral reactions, such as blinking and backward head movements, to looming stimuli from birth. In contrast, they do not react to zooming stimuli. These reactions are thought to be an adaptation to potentially threatening approaching objects; in fact, zooming stimuli signaling receding objects do not elicit such reactions (e.g., Ball and Tronick 1971; Náñez Sr 1988). More recently, studies using looking time measures showed that newborns discriminate between visual displays depicting different motion trajectories in the depth plane, showing a preference for those approaching their peripersonal space (Orioli, Bremner, and Farroni 2018a; Orioli, Filippetti, et al. 2018b). In adults, visual looming stimuli enhance the detection of tactile (Cléry et al. 2015; Kandula et al. 2015) and visuo‐tactile (Geers et al. 2024) stimuli, a multisensory facilitation effect due to a combination of attentional factors and contact anticipation. Building on this, Macchi Cassia and colleagues (de Hevia et al. 2017) speculated that the alerting effect engendered by looming perception might facilitate processing the increasing order information embedded in such stimuli. This, in turn, could generalize to an advantage for increasing order even in the absence of objective looming cues, as observed in studies reporting the asymmetry for size (Macchi Cassia et al. 2012) and numerical (de Hevia et al. 2017) order at 4 months.

### 1.1 The Current Study

In the current study, we explored the developmental origins of the asymmetry signature in order processing by focusing on newborns' performance. We examined the ability of few‐day‐old newborns to detect and represent ordinal relations in non‐symbolic numerical sequences that lack looming or zooming visual cues.

Research has shown that 2–3‐day‐old newborns can discriminate between large numerosities (e.g., 4 vs. 12) when the ratio is sufficiently large (1:3) (Coubart et al. 2014; Izard et al. 2009). They can also generalize this ability across different types of magnitudes (number, size, time; de Hevia et al. (2014)) and sensory modalities (auditory and visual; Coubart et al. 2014; Izard et al. 2009). Izard et al. (2009) familiarized participants with auditory sequences containing a fixed number of syllables. They then tested them with arrays of visual items with numerosity that either matched or mismatched the familiarized soundstream, playing concurrently in the background. Newborns looked longer at arrays with numerosity matching the soundstream than at those differing in number by a 1:3 ratio. Using a modified version of the same task, de Hevia et al. (2014) showed that, after familiarization with a short or long stream of beats paired with a corresponding small or large visual object, newborns reacted to a simultaneous increase (or decrease) in both beat number/duration and object size, but not when the magnitudes changed in opposite directions (e.g., increasing beat number/duration paired with decreasing object size).

These and other similar studies indicate that the ability to compare and discriminate numerical differences is present at birth and, as in other animal species and older infants (see review by Cordes and Brannon 2008), this ability is modulated by numerical ratio. However, no research has investigated whether human newborns can appreciate not only the difference but also the direction (increasing or decreasing) of numerical changes, that is, if they are able to perform ordinal computations on numerical representations. It is important to emphasize that existing evidence on newborns' numerical discrimination does not always require reliance on memory‐based representation of perceived quantity. This is because, in the majority of the studies, the stimuli were perceptually available as they were presented simultaneously, reducing memory load while allowing for direct comparison (Coubart et al. 2014; de Hevia et al. 2014). Therefore, the ability of newborns to maintain representations with approximate numerical content in working memory, a prerequisite for ordinal computations, remains unknown.

The current experiments aimed to provide direct evidence for the detection of numerical order in newborn infants by testing their ability to generalize the order structure learned from sequentially presented numerical displays to new numerosities, discriminating it from a reversed order. We adapted the method used in previous studies with older infants (de Hevia et al. 2017; Picozzi et al. 2010; Suanda et al. 2008), where redundant cues to ordinality were added within and across sequences. Research suggests that younger infants require multiple perceptual cues to succeed at tasks that older infants can solve with a single cue (e.g., Bahrick and Lickliter 2004; Jordan et al. 2008; Kirkham et al. 2007). In line with this, research in the numerical domain has shown that synchronized audiovisual presentations promote infants' representational precision, leading to better numerical discrimination (Jordan et al. 2008). Additionally, increasing stimulus variability during learning can enable infants to encode more abstract features of the stimuli (e.g., Perry et al. 2010). Taking insight from this literature, and following the methods of Picozzi et al. (2010) and de Hevia et al. (2017), in the current study we designed and presented the numerical displays to emphasize the target order (increase or decrease) both within and between sequences. In all cases, the numerosities differed by a 1:3 ratio. For instance, in the increasing habituation condition, not only did numerosities increase within each sequence (e.g., 4, 12, 36), but consecutive sequences also progressed in a fixed increasing order (first trial: 4, 12, 36; second trial: 6, 18, 54; third trial: 7, 21, 63).

In Experiment 1, we examined whether newborns could extract increasing order from numerical sequences, building on de Hevia's (2017) positive evidence in 4‐month‐olds. To ensure that non‐numerical continuous variables did not provide any cue to the ordinal relations, cumulative surface area and contour length were kept constant within the sequences by varying item size and shape inversely to number.

In Experiment 2, newborns' ability to extract both increasing and decreasing order was tested under facilitating conditions, by adding redundant non‐numerical quantitative cues to the numerical sequences. Item size covaried with numerosity within each sequence, resulting in positively correlated changes in the continuous dimensions of cumulative surface area, contour length, and density. This procedure was modeled after Suanda et al. (2008), who reported positive evidence of numerical order discrimination in 9‐month‐old infants for both increasing and decreasing sequences in the presence of multiple convergent quantitative cues but not when number, item size, or cumulative surface area were available in isolation. Newborns have been shown to generalize discrimination of number to area (de Hevia et al. 2014), suggesting that both dimensions are represented under a common format, available from birth (for reviews, see Bonn and Cantlon 2017; de Hevia 2016). Therefore, we hypothesized that by providing redundant cues that integrate both numerical and continuous dimensions, we might facilitate newborns' detection, generalization, and discrimination of numerical order, potentially by promoting access to a more abstract representation of number magnitude. If these redundant quantitative cues help newborns process ordinal relations, similar to 9‐month‐olds (Suanda et al. 2008), in Experiment 2 we expected them to successfully discriminate order reversal after habituation to both increasing and decreasing sequences. However, successful discrimination following habituation to increasing sequences, coupled with a failure to discriminate after habituation to decreasing sequences, similar to 4‐month‐olds (de Hevia et al. 2017), would provide compelling evidence that the asymmetry signature in ordinal processing may have deep evolutionary roots. Finally, to isolate the influence of non‐numerical cues in Experiment 2's findings, Experiment 3 investigated newborns' ordinal discrimination abilities for size‐based sequences composed of single shapes that differed in size and contour length in the absence of any numerical cue.

## 2 Experiment 1. Newborns' Detection of Increasing Numerical Order

### 2.1 Methods

#### 2.1.1 Participants

The final sample included 22 healthy full‐term newborn infants (11 females) (mean age = 41 h; range = 15–98 h; mean weight = 3471 g; range = 2540–4400 g; APGAR score of 10 at 5 min). For both Experiment 1 and Experiment 2, the sample size was estimated based on de Hevia et al. (2017, Exp. 2). Because that study did not report the specific effect size for the post hoc comparison between familiar and novel test trials in the increasing habituation condition, we set the sample size of Experiment 1 to match that of the increasing habituation condition of Experiment 2 (see the participant section of Experiment 2 for a detailed description of the parameters used for sample size calculation). Nine additional infants were tested and excluded from the final sample because of drowsiness/fussiness (*N* = 7), equipment failure (*N* = 1), and looking times in at least one test trial shorter than 2 s (*N* = 1). This attrition rate is within the expected range for looking‐time studies with 24‐h‐old newborns, particularly when using a sequential presentation procedure with multiple test trials (e.g., de Hevia et al. 2014, 2025; Izard et al. 2009). Infants were recruited at the maternity ward of the Mangiagalli Clinic in Milan between 2018 and 2022, and tested when in an awake and alert state. The study was approved by the Milano Area 2 Ethical Committee, Fondazione IRCCS Ca' Granda Ospedale Maggiore Policlinico (ID: 694; Approval No. 624_2018), and parents signed an informed written consent before testing began.

#### 2.1.2 Stimuli

**FIGURE 1:**
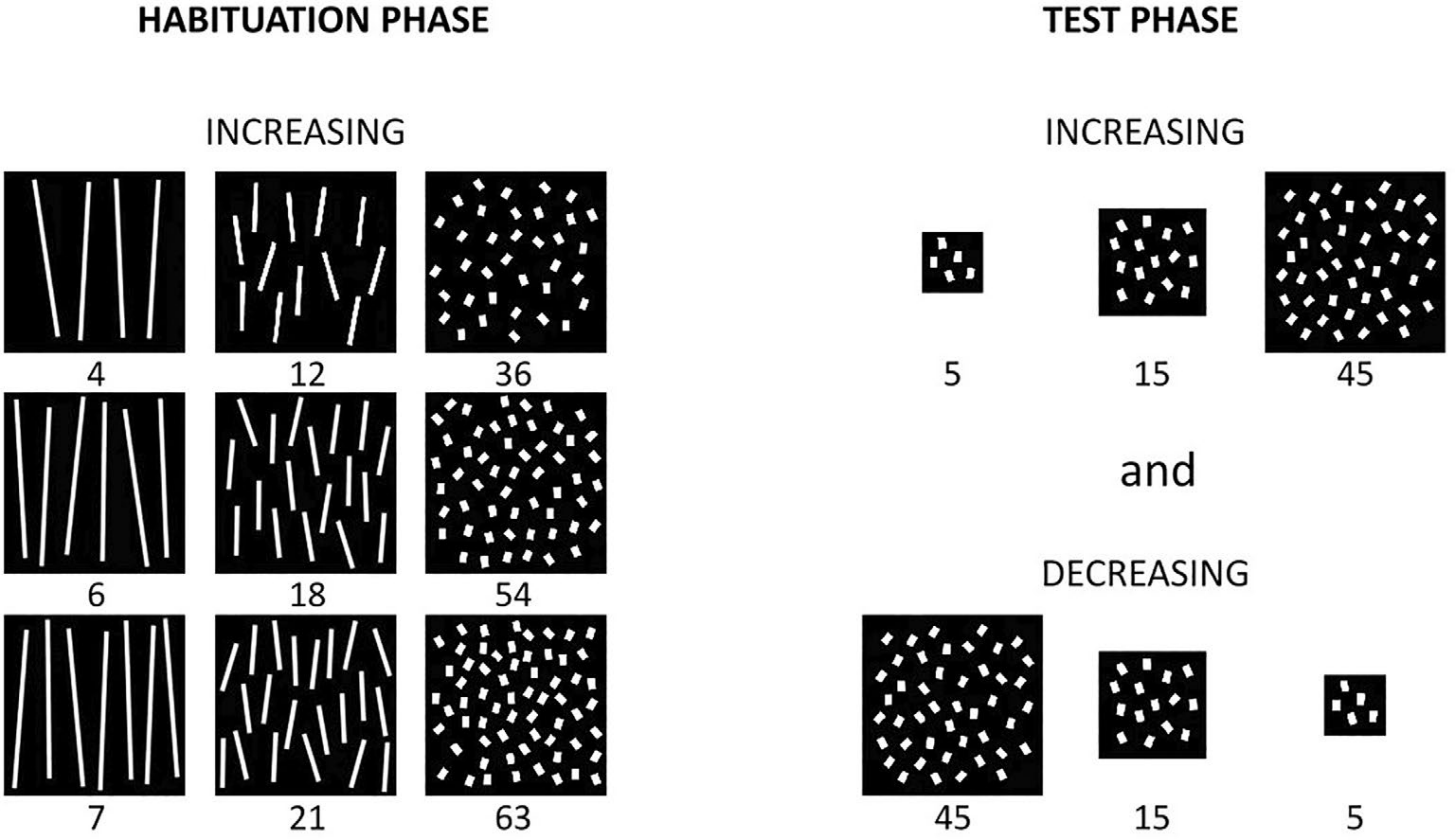
The four sets of stimuli presented in Experiment 1. In the habituation stimuli (left), item size was inversely correlated to number according to a 1:3 ratio, and virtual display area remained constant. This maintained constant cumulative surface area and contour length while allowing density to covary with numerosity. The test stimuli (right) kept item size constant but increased the virtual display area threefold as the number of items increased. This resulted in a threefold increase in cumulative surface area and contour length, while density remained constant across numerosities.

Stimuli were sequences of three visual displays generated using E‐Prime 1.0 software. Each display contained a fixed number of white, rectangular items arranged randomly within a virtual black area. The black area appeared on a black background, creating a seamless presentation where the outline of the item‐containing area was indistinguishable (Figure 1). Three sets of stimuli were used for the habituation phase and one for the test phase. The number of items contained in each sequence differed by a 1:3 ratio. The three habituation sets contained 4‐12‐36, 6‐18‐54, and 7‐21‐63 items, while the test set contained 5‐15‐45 items. In the habituation sets, non‐numerical continuous variables were controlled by keeping cumulative surface area and contour length constant within each set. To achieve this, item size was inversely correlated to number according to a 1:3 ratio, resulting in approximate sizes of 132, 44, and 14.7 cm^2^ for the smaller, medium, and larger items respectively. The virtual area of each display was kept constant at 800 × 800 pixels, so that number covaried with density. For test displays, item size was kept constant at the size of the smallest items from habituation (14.7 cm^2^), so that the cumulative surface area was positively correlated with number. Virtual display area increased proportionally (i.e., tripled) to the number of items, so that density was held constant across numerosities. This way, the continuous, non‐numerical variables that varied during habituation were held constant during the test, and vice versa.

#### 2.1.3 Apparatus and Procedure

Methods were modeled after de Hevia et al. (2017) and Macchi Cassia et al. (2012), where 4‐month‐olds were tested in their abilities to represent ordinal information for numerical and size‐based sequences, respectively. Newborns were tested in a visual habituation task using an infant‐controlled procedure. During habituation, the three habituation stimulus sets were cycled until the infant reached the habituation criterion, which specified that from the fourth trial onwards, the sum of looking times in three consecutive trials is to be equal to or less than 50% of the sum of looking times in either the first three trials or the three preceding trials in which 50% of the fixation times exceeded the initial criterion (Colombo and Mitchell 2009). They were presented in a fixed order, from the smallest to the largest (i.e., 4‐12‐36, 6‐18‐54, 7‐21‐63), so as to provide convergent cues to ordinality both within and between habituation sequences. Following habituation, infants viewed four test trials with new numerical values alternating increasing and decreasing order; test order was counterbalanced across participants.

Infants sat on the experimenter's lap, approximately 30 cm from the stimulus presentation monitor (27″ screen size, 1920 × 1080 pixel resolution, 60 Hz refresh rate). To avoid distractions, the lights in the room were turned off, and black panels were placed on the sides of the screen. A video camera positioned just above the stimulus presentation monitor sent the live image of the newborn's face to another monitor, allowing a second experimenter, blind to the test order condition, to code online newborns' looking times through E‐Prime software. The newborn's gaze was also recorded via a Mini‐Dv digital recorder so that a second observer could code offline the looking times towards the stimuli. Another monitor, showing the image of the newborn's face, was placed just above the stimulus presentation screen so that the experimenter holding the baby could manage to have his/her head aligned to the center of the screen. The parents were present during the entire experimental session and could interact with the baby in case of crying or discomfort.

**FIGURE 2:**
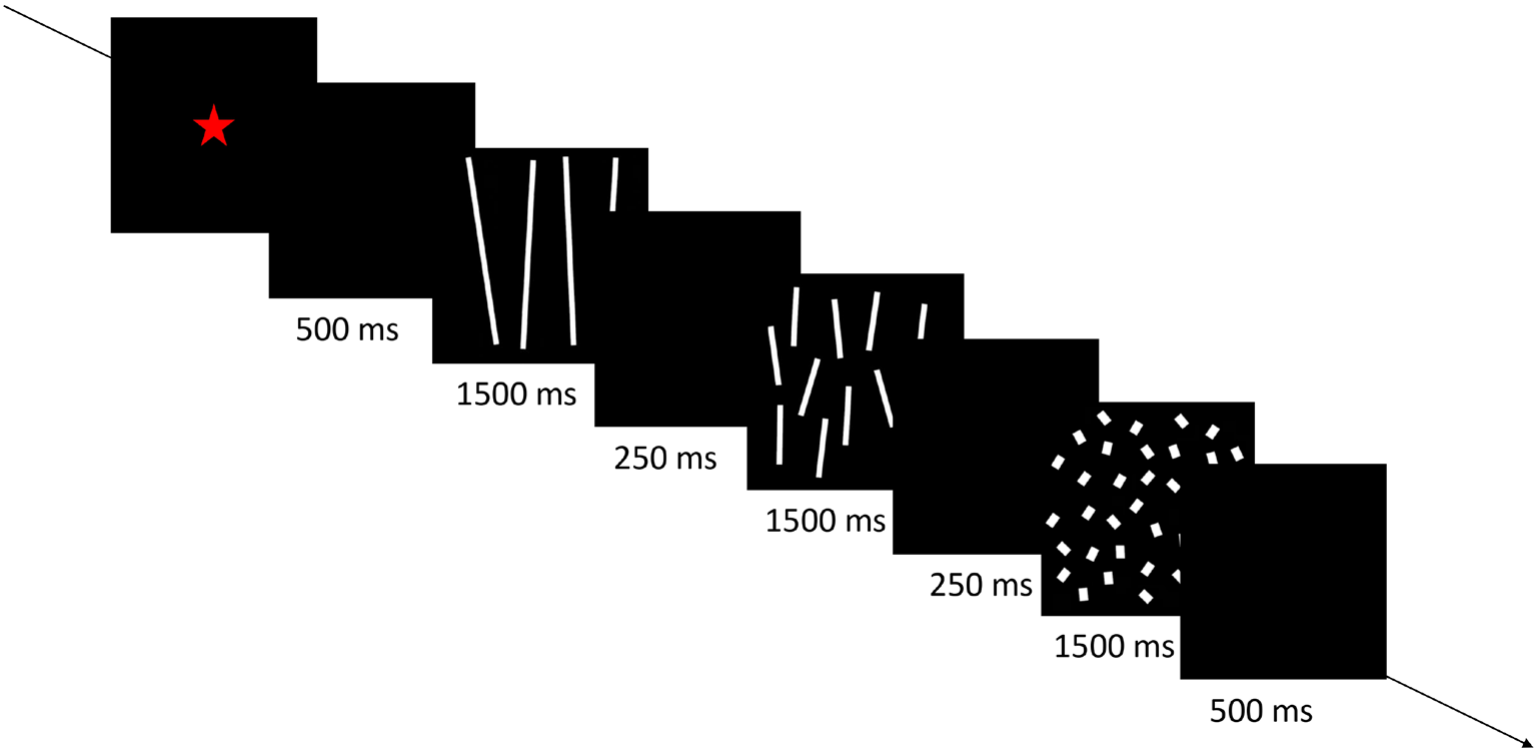
Illustration of the stimulus presentation trial structure used in Experiments 1, 2, and 3.

Each trial began as soon as the newborn fixated on a red flickering star appearing in the center of the screen. The trial consisted of a repeating cycle (6250 ms in total) composed of a black screen (500 ms) followed by the three numerical displays appearing on a black background, and then by a gray screen (500 ms). Each numerical display remained on the screen for 1500 ms and was followed by a 250 ms blank (Figure 2). The trial continued until the newborn met a minimum looking time of 500 ms and ended when the newborn looked away continuously for 2 s or looked for a maximum of 120 s. The three habituation sets were presented in a fixed order until the infant viewed 18 trials or met the habituation criterion. Following habituation, infants saw four test trials in which a sequence composed of new numerosities arranged in the familiar, increasing order was presented alternated with the same sequence arranged in a novel, decreasing order, with half of the newborns seeing the novel test order first.

### 2.2 Data Reduction and Analysis

Newborns' looking times during test trials were coded offline from the video recordings by a second observer, who was blind to the test order condition the newborn was assigned to. Offline coding was performed frame‐by‐frame using VirtualDub software at a resolution of 25 frames per second (40 ms). Given the inherent challenges of online coding eye gaze in newborns, particularly when their eyes are swelling or not wide open, and the need for flexibility during online coding (e.g., infant yawning or sneezing), a third observer coded test trials offline where the two initial coders' judgments diverged by more than 5 s (26% of all trials). Statistical analyses were based on the average of the two closest measurements for each test trial, which could originate from either online and offline coding or from offline coding alone (see Bonn et al. 2019 and de Hevia et al. 2014 for a similar procedure). The Pearson correlation between the two measurements, as assessed on total fixation times on each of the four test trials, was *r* = 0.98.

Statistical analyses were performed using *R* (R Core Team, 2024). We adopted two parallel data analysis strategies. In line with best practices for the statistical treatment of infant looking‐time data (Csibra et al. 2016), we log‐transformed the data before applying parametric statistical methods (ANOVA and *t*‐tests). However, recognizing that log‐transformation can potentially obscure meaningful patterns in the data while addressing non‐normality and heteroscedasticity, we also analyzed raw data after checking for normality using the Shapiro–Wilk test. When the test indicated a significant deviation from normality, we analyzed the raw data using non‐parametric tests like Aligned Rank Transform (ART) analysis performed through the *ARTool* package in R, Mann–Whitney, and Wilcoxon tests.

### 2.3 Results

**FIGURE 3:**
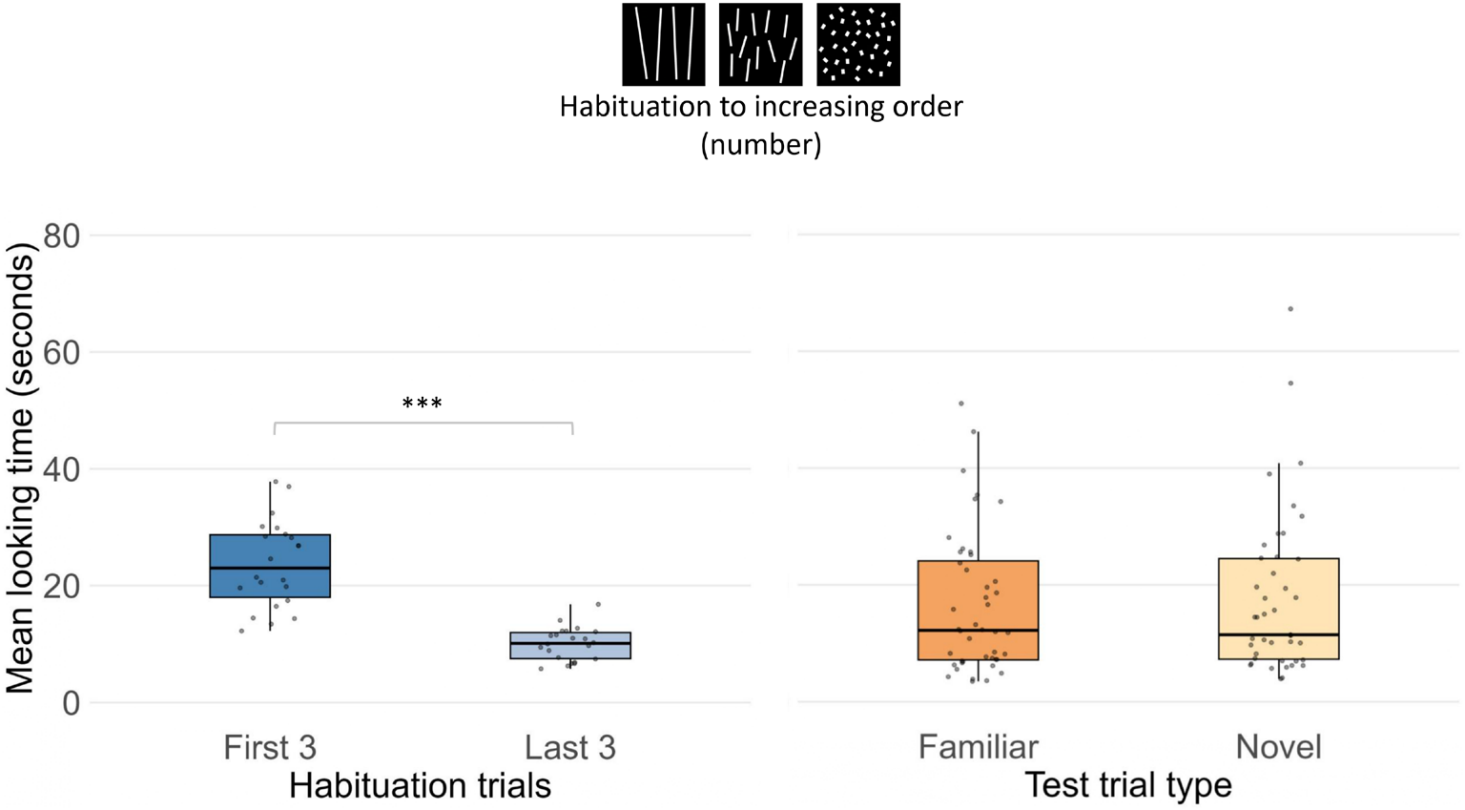
Mean total looking time in Experiment 1 for the first three and last three habituation trials and for familiar and novel test trials. Black lines represent the median, the boxes indicate the interquartile range (IQR), and dots show the individual data points. ***p < 0.001.

Newborns required an average of 8.27 (SD = 2.93) trials and 141 s (SD = 50.8) of total looking time to habituate (Table 1). A paired‐samples *t*‐test confirmed the presence of a significant decline in mean looking time from the first three habituation trials (*M* = 23.7 s, SD = 7.48) to the last three (*M* = 10 s, SD = 2.83), with both log‐transformed, *t*(21) = 17.1, *p* < 0.001, Cohen's *d* = 3.65, and raw data, *t*(21) = 11.11, *p* > 0.001, Cohen's *d* = 2.37 (Figure 3).

**TABLE 1:** Descriptive statistics of the raw dependent variables measured during habituation in the three experiments, and statistical significance of the comparison between the increasing and decreasing habituation order conditions tested in Experiments 2 and 3.

Variables	Increasing		Decreasing		Statistical significance
	Mean	SD	Mean	SD	*p*
Experiment 1					
Total LTs (s)	141	50.8			
No. of trials	8.27	2.93			
Proportional decrement	0.56	0.11			
Experiment 2					
Total LTs (s)	113.2	41.45	137.82	53.88	0.33
No. of trials	6.95	1.89	8.59	3.25	0.028
Proportional decrement	0.64	0.12	0.53	0.18	0.017
Experiment 3					
Total LTs (s)	118.65	54.82	146.42	93.44	0.4
No. of trials	8.41	2.84	7.73	1.83	0.68
Proportional decrement	0.45	0.3	0.3	0.65	0.94

To determine whether in the test newborns were able to generalize to novel numerosities the familiar numerical order they have been habituated to and discriminate it from the novel reversed order, total looking times during test trials were analyzed. A three‐way Analysis of Variance (ANOVA) with first test trial (familiar vs. novel) as the between‐subjects factor, and test trial pair (first vs. second) and test trial type (familiar vs. novel order) as within‐subjects factors, revealed no main effects or interactions (*p*s > 0.110). As data were not normally distributed (*W*s < 0.91, *p*s < 0.05), we performed a nonparametric ART analysis on raw looking times using first test trial, test trial type, and pair as fixed effects and a random intercept for participants, which also revealed no significant effects (all *p*s > 0.11) (Figure 3).

Binomial tests on individual infant data revealed no significant difference in the number of infants who looked longer at the novel order compared to those looking longer at the familiar one (binomial test: 13 vs. 9, *p* = 0.26, one‐tailed).

### 2.4 Interim Discussion

Results provided no evidence that newborns could discriminate between increasing and decreasing numerical order, nor that they could generalize the increasing order embedded in the habituation numerical sequences to novel numerosities at test. Although newborns' looking times to the decreasing (novel) order at test (*M* = 19.2 s, SD = 12.56) were slightly longer than their looking times to the increasing (familiar) order (*M* = 16.3 s, SD = 10.08), they were not statistically different. A similar trend emerged from the analyses on individual infants, showing the number of newborns who showed a novelty preference at test did not differ significantly from that of those who looked longer at the familiar order.

One possible explanation for the lack of ordinal discrimination is that newborns may have habituated to the numerical displays but failed to grasp the ordinal direction of the numerical changes within and between the triplets due to limitations in their ability to perform ordinal computations. Alternatively, the representation of increasing order established during habituation might have been too weak to support discrimination and generalization. Inspired by evidence suggesting that combining numerical and non‐numerical cues improves infants' representational precision, promoting ordinal discrimination in older infants (Suanda et al. 2008), we hypothesized that this approach could also benefit newborns. To explore this possibility, two new groups of newborns were tested in Experiment 2, incorporating non‐numerical quantitative cues to ordinality into both increasing and decreasing sequences.

## 3 Experiment 2. Newborns' Detection of Numerical Order in the Presence of Co‐Varying Non‐Numerical Quantitative Cues

In Experiment 2, newborns were habituated to either increasing or decreasing numerical sequences, and were tested with new sequences displaying both the familiar and the novel orders in alternation. Within each sequence, number covaried with cumulative surface area, contour length, and density, providing cumulative convergent cues to ordinality.

### 3.1 Methods

The methods were the same as in Experiment 1 except as follows.

#### 3.1.1 Participants

Forty‐four healthy full‐term newborns (23 girls) were included in the final sample (mean age = 43 h, range = 13–89 h; mean birth weight = 3425 g; range = 2700–4510 g; APGAR score of 10 at 5 min). Seventeen additional infants were tested and excluded from the sample because they failed to complete testing due to drowsiness or fussiness (*N* = 11; 5 in the increasing and 6 in the decreasing habituation condition), equipment failure (*N* = 1), experimenter error (*N* = 1), and looking times less than 2 s (*N* = 4). Sample size was estimated based on de Hevia et al. (2017, Exp. 2). An a priori power analysis performed using G‐Power revealed that a total sample size of at least 14 participants (7 per group) would have provided enough power (0.95 at α = 0.05) to detect an interaction between habituation order (increasing, decreasing; between groups) and test ordinal direction (familiar, novel; within group) at the same large effect size (*f* = 0.53). Outputs of the sample size calculation are available at https://osf.io/aezq6/?view_only=18aea219c7eb4c368f6ce9503e128bea. Recruitment followed the same procedures as Experiment 1, and infants were tested if awake and in an alert state. Half were randomly assigned to either the increasing or decreasing habituation condition; there were no significant group differences in gestational age, birth weight, or age at testing (all *p*s > 0.31).

#### 3.1.2 Stimuli

**FIGURE 4:**
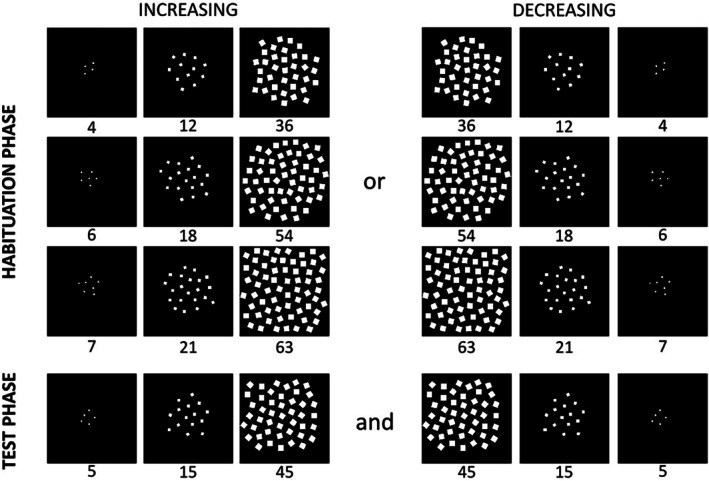
The four sets of stimuli presented to newborns in the increasing (left) and decreasing (right) order condition in the habituation (top) and test (bottom) phase of Experiment 2. In both habituation and test phases, item size increased or decreased proportionally with number according to a 1:3 ratio, and virtual display area remained constant. This resulted in a ninefold difference in cumulative surface area and contour length across numerosities and covarying density.

Stimuli were random configurations of white squares on a black background that varied in numerosity, cumulative surface area, and density. As in Experiment 1, there were three sets of habituation stimuli and one set of test stimuli, and each set contained three displays (Figure 4). The numerosities used in both habituation and test were identical to Experiment 1. However, in all sets, item size was positively correlated with number, as it increased (or decreased) three‐fold between displays in each set (4, 12, 36 cm^2^). Since both number and item size increased or decreased by a factor of three, the cumulative surface area of the displays consequently increased ninefold within each stimulus set (e.g., 16, 144, 1296 cm^2^ for the numerosities 4, 12, 36). In all sets, density also varied systematically with the items' number, because the virtual area of each display was kept constant at 800 × 800 pixels. The items in the smallest displays measured 0.65 × 0.65 cm (after Simion et al. 2008), subtending a visual angle of approximately 1.2° at a viewing distance of 30 cm (corresponding to 0.83 cycles per degree, cpd). Given that newborn acuity is typically around 1 cycle per degree (cpd) (e.g., Acerra et al. 2002; Atkinson et al. 1979; Banks and Bennett 1991; Brown and Yamamoto 1986; Brown et al. 2018), even these smaller displays were well within the newborn visual acuity threshold.

#### 3.1.3 Procedure

Stimuli were presented as in Experiment 1 (Figure 2). During habituation, half of the infants viewed increasing sequences and the other half viewed decreasing sequences. Infants were randomly assigned to each habituation condition. Like in Experiment 1, the three numerical displays within each set were presented in a consistent fixed order: from the smallest to the largest for the increasing condition and from the largest to the smallest for the decreasing condition. All infants viewed four test trials with new numerical displays alternating increasing and decreasing sequences, and test order counterbalanced across participants.

Following de Hevia et al. (2014), a third observer independently coded test trials offline where the two initial coders' judgments differed by more than 5 s (23% of all trials). Similar to Experiment 1, statistical analyses were based on averages of the two closest values for each trial. Intercoder agreement between the two measurements was *r* = 0.98, as assessed by Pearson's correlation on total fixation times on each of the four test trials.

### 3.2 Results

A repeated‐measure ANOVA with habituation order (increasing vs. decreasing) as a between‐subjects factor and habituation trials (first three vs. last three) as a within‐subjects factor on average log‐transformed looking times revealed a significant main effect of habituation trials, *F*(1,42) = 310.7, *p* < 0.001, *η* ^2^_p_ = 0.881, due to longer looking times on the first three habituation trials (M = 23.15 s, SD = 9.94) than on the last three trials (M = 8.98 s, SD = 4.34). There was also a significant Habituation trials × Habituation order interaction, *F*(1) = 5.29, *p* = 0.027, *η* ^2^_p_ = 0.112. As data for the last three habituation trials were not normally distributed (*W*s < 0.88, *p*s < 0.009), we also performed an ART analysis on raw looking times using habituation order and habituation trial as fixed effects, and a random intercept for participants. Results confirmed those emerged from the parametric analysis on log‐transformed data, showing a main effect of habituation trial, *F*(1,42) = 294.93, *p* < 0.001, and a marginally significant Habituation trial × Habituation order interaction, *F*(1,42) = 3.77, *p* = 0.059. Although the decrement in average looking times between the first three and last three habituation trials was significant for both habituation order conditions (*p*s < 0.001), the mean difference between the first and the last three habituation trials was slightly larger for the increasing condition (*M* = 0.46, SE = 0.033) than for the decreasing one (*M* = 0.36, SE = 0.033), suggesting more efficient habituation to increasing order.

To follow up these interactions and further explore the efficiency of newborns' habituation to the two orders, we also contrasted the proportional looking time (LT) decrement (i.e., mean raw LTs in the first three trials‐mean raw LTs in the last three trials/mean raw LTs in the first three trials) during habituation to the increasing sequences (*M* = 0.64, SD = 0.12) and the decreasing ones (M = 0.53, SD = 0.18). While the resulting variable was not normally distributed (*W* = 0.9, *p* = 0.032), the comparison was significant, Mann–Whitney *U* = 141, *p* = 0.017, Rank biserial correlation = 0.42 (two‐tailed), suggesting more efficient learning of increasing than decreasing order. Accordingly, despite similar total habituation time across the two habituation order conditions (increasing *M* = 113.2 s, SD = 41.45 vs. decreasing: *M* = 137.82 s, SD = 53.88), *t* > 1 with both log‐transformed and raw data (two‐tailed), participants required a smaller number of trials to habituate to the increasing sequences (*M* = 6.95, SD = 1.89) compared to the decreasing ones (*M* = 8.59, SD = 3.25) (*W*s < 0.8, *p*s < 0.001), Mann–Whitney *U* = 153, *p* = 0.028, Rank biserial correlation = 0.37 (two‐tailed).

**FIGURE 5:**
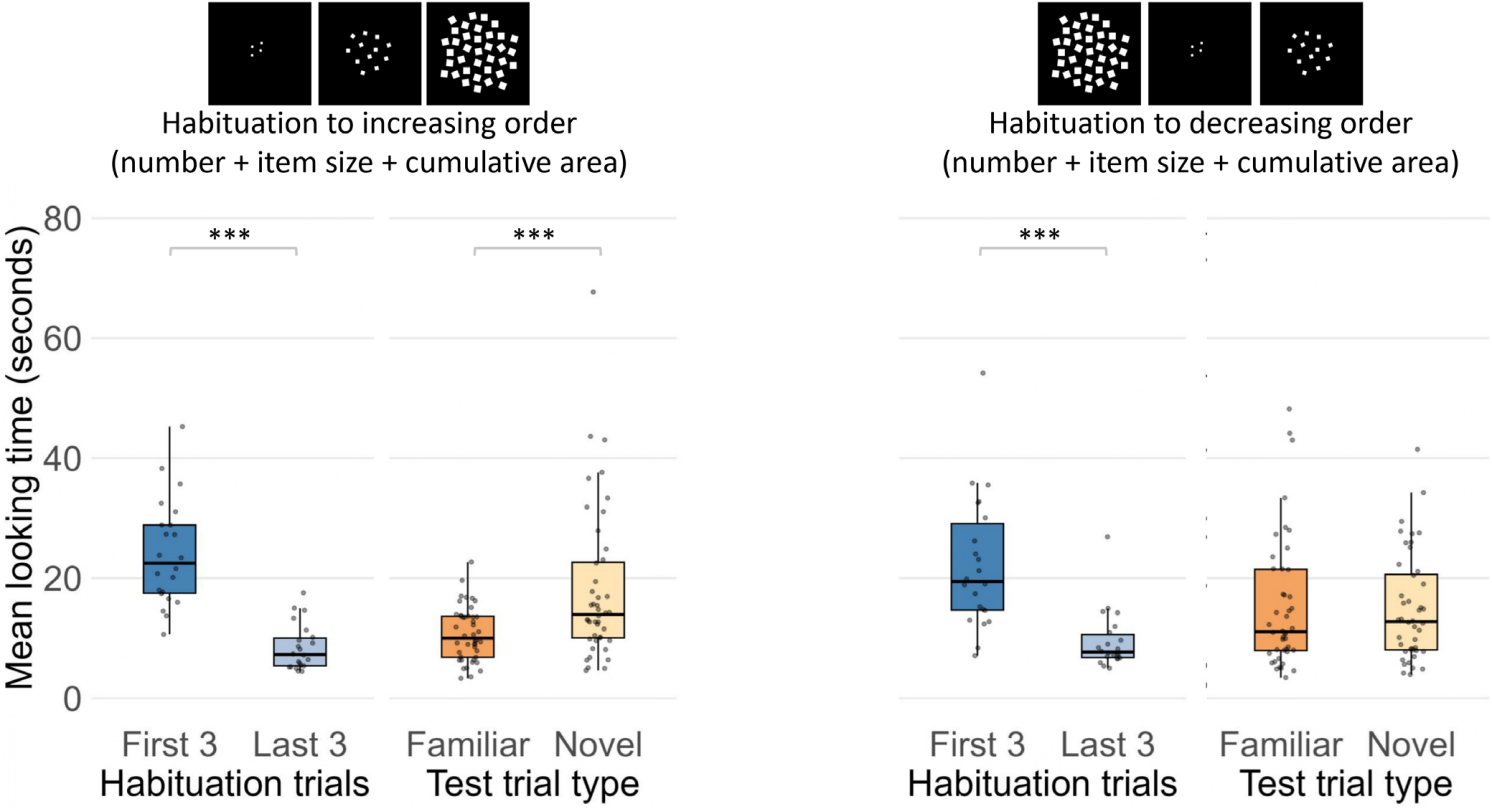
Mean looking time in Experiment 2 to the first three and last three habituation trials and to familiar and novel test trials for newborns in the increasing (left) and decreasing (right) habituation order conditions. Black line represents the median, the box indicates the interquartile range (IQR), dots show the individual data points. ***p < 0.001.

To determine whether participants could discriminate familiar order from the novel order at test, a four‐way ANOVA was performed on log‐transformed total looking times during test trials. The ANOVA included habituation order (increasing vs. decreasing) and first test trial (familiar vs. novel) as between‐subjects factors and test trial pair (first vs. second) and test trial type (novel vs. familiar order) as within‐subjects factors. There was a significant main effect of test trial type, *F*(1,40) = 7.44, *p* = 0.009, *η* ^2^_p_ = 0.157, which was qualified by a significant Test trial type × Habituation order interaction, *F*(1,40) = 6.22, *p* = 0.017, *η* ^2^_p_ = 0.135. Newborns looked overall longer to the novel (*M* = 16.51 s, SD = 8.28) than to the familiar (*M* = 13.05 s, SD = 6.94) order across the two test trials pairs, but this was true only for newborns habituated to the increasing order (familiar order: M = 10.7 s, SD = 3.22, vs. novel order: *M* = 18.2 s, SD = 8.56; *p* = 0.004, Bonferroni corrected), not for those habituated to the decreasing order (familiar order: *M* = 15.3 s, SD = 8.78 vs. novel order: *M* = 14.9 s, SD = 7.87; *p* = 1.0, Bonferroni corrected). No other effects or interactions were significant, all *p*s > 0.245 (Figure 5). Similar results were obtained from the ART analysis on raw looking times (*W*s < 0.8, *p*s < 0.03), using habituation order, first test trial, test trial pair, and test trial type as fixed effecs, and a random intercept for participants. There were a Test trial type, *F*(1,120) = 4.27, *p* = 0.041 and a Test trial type × Habituation order interaction, *F*(1,120) = 4.09, *p* = 0.045: newborns habituated to the increasing order showed a significant novelty preference, *t*(120) = 21.3, *p* = 0.03, while those habituated to the decreasing order, *t*(120) = 6.68, *p* = 0.5, did not.

Analyses of individual infant data revealed that this novelty preference was shown by 17 of the 22 newborns (binomial test: *p* = 0.008, one‐tailed) assigned to the increasing habituation condition, while it was shown by only 9 newborns out of the 22 (binomial test: *p* = 0.86, one‐tailed) assigned to the decreasing habituation condition.

#### 3.2.1 Comparison Across Experiment 1 and Experiment 2

The combined findings from Experiment 1 and Experiment 2 suggest that manipulating the amount of quantitative cues available in the increasing ordinal sequences impacted newborns' performance. This manipulation likely enhanced the saliency of the magnitude changes within and between the sequences, thereby improving the precision and abstraction of the ordinal representation formed during habituation.

This was confirmed by the comparison of newborns' performance during habituation and test trials of Experiment 1 with the performance of newborns in the ascending order condition of Experiment 2. An unpaired *t*‐test performed on log‐transformed data showed that total looking time to habituate was longer in Experiment 1 (*M* = 141 s, SD = 50.8) compared to Experiment 2 (*M* = 113 s, SD = 41.4), *t*(42) = 2.12, *p* = 0.04, Cohen's *d* = 0.064. A similar trend emerged from the nonparametric Mann–Whitney test on raw total habituation times (*W* = 0.91, *p* = 0.04), *U* = 161, *p* = 0.059, albeit marginally significant, and for the proportional decline in looking times across the first three and the last three habituation trials, (*W* = 0.8, *p* < 0.001) *U* = 150, *p* = 0.03, Rank biserial correlation = 0.38, which was steeper in Experiment 2 (*M* = 0.64, SD = 0.12) compared to Experiment 1 (*M* = 0.56, SD = 0.11).

Newborns' test performance across the two studies was contrasted by comparing novelty preference scores, calculated as the ratio between raw total looking times towards the novel trials and the combined looking times to familiar and novel trials (Bulf et al. 2021). The comparison did not reach statistical significance, *t*(42) = 1.92, *p* = 0.061, Cohen's *d* = −0.58. One‐sample *t*‐tests were then used to compare the novelty preference scores within each experiment to the chance level (50%). For Experiment 1, no significant novelty preference was found (*M* = 0.51, SD = 0.04), *t*(21) = 1.1, *p* = 0.28, whereas the novelty preference was reliable for Experiment 2 (*M* = 0.53, SD = 0.04), *t*(21) = 4.4, *p* < 0.001, Cohen's *d* = 0.94.

### 3.3 Interim Discussion

Overall, group analyses and those from individual infants concurred in showing that newborns in Experiment 2 successfully discriminated a reversal in ordinal direction at test after being habituated to increasing sequences of discrete elements varying in numerosity as well as continuous magnitudes. Newborns exhibited longer looking times to the novel test trials depicting decreasing order. From such a novelty preference, we can infer that newborns have not only distinguished between the two ordinal directions but have also generalized the familiarized order to the novel magnitudes presented in test trials. This indicates that they successfully extracted and represented the ordinal relations embedded in the habituation sequences.

Importantly, this same ability was not observed following habituation to decreasing sequences. Newborns failed to show any preference for either the novel or familiar test sequences after habituation to decreasing order. Furthermore, habituation to decreasing sequences required more trials and resulted in a shallower decline in looking times compared to increasing sequences. This suggests that newborns formed a more stable representation of the ascending relations between increasing values, which likely facilitated generalization to new values at test.

These findings align with those from 4‐month‐old infants, who exhibited a similar asymmetry in processing numerical (de Hevia et al. 2017) and size‐based (Macchi Cassia et al. 2012) sequences. In the case of size ordering, 4‐month‐olds were shown to be blind to decreasing order, as they failed to discriminate a sequence in which the size of a shape consistently decreased from a sequence where the same size values changed non monotonically (i.e., randomly) (Macchi Cassia et al. 2012, Experiment 1B). Our results do not tell us whether this applies also to newborns. Therefore, the question of whether our participants simply represented decreasing sequences as a collection of different numerical sets, without encoding the directionality of the magnitude changes, or whether the representation they built during habituation was just too blurred to support generalization to new test values, remains open. Regardless, the convergence of our findings with those reported for 4‐month‐old infants (de Hevia et al. 2017; Macchi Cassia et al. 2012) supports the idea that the observed ascending bias in order processing, favoring increasing over decreasing sequences, is present from birth, at least when stimuli include both numerical and non‐numerical quantitative cues to ordinality.

While Experiment 1 did not reveal an ascending bias for numerical order processing in newborns, the results of Experiment 2 remain inconclusive regarding the specificity of this bias to size‐based order processing. The stimuli used in Experiment 2 allowed infants to use either the surface area of individual items or the cumulative surface area of all items included in the numerical displays as a basis for their order discrimination, as both dimensions varied in the same direction. Although newborns can discriminate 1:3‐ratio changes in numerosity (Coubart et al. 2014; Izard et al. 2009), our findings from Experiment 1 indicate that they cannot perform ordinal operations based solely on such changes. Nonetheless, it is likely that numerical changes played a crucial role in boosting the processing of increasing order in Experiment 2 for a number of reasons. First, evidence from older infants indicates that they may not represent the size of a single element very precisely when it is part of a homogeneous set like those used in the current study (Libertus et al. 2014). For example, while 6‐month‐old infants can discriminate between different sizes of a single element with a 1:2 ratio (Brannon et al. 2006a, 2006b), 7‐month‐olds require at least a 1:4‐ratio change when the element is presented within a set of homogeneous elements (Cordes and Brannon 2011). This makes it unlikely that infants relied solely on changes in the size of individual elements to detect the increasing or decreasing order in our study. Second, recent evidence in adults suggests that numerosity and cumulative area are perceived holistically as integral dimensions, where a change in one dimension causes a perceived change in the other (Aulet and Lourenco 2021). It is possible that this perceptual phenomenon was amplified in Experiment 2 as both number and element size increased or decreased by a factor of three, thus resulting in a substantial ninefold change in the cumulative surface of the displays.

In light of this, it seems unlikely that newborns in Experiment 2 based their representation of increasing order solely on 1:3‐ratio changes in the size of individual elements within the numerical displays. To isolate the influence of numerical and continuous quantitative cues in Experiment 2's findings, in Experiment 3 we investigated newborns' ability to discriminate ordinal direction in size‐based sequences composed of single shapes differing in surface area and contour length.

## 4 Experiment 3. Newborns' Detection of Size‐Based Order

Experiment 3 investigated whether newborns can extract ascending (and descending) ordinal relations based solely on physical size and contour length when no numerical cues are available. Newborns were habituated to increasing or decreasing size‐based sequences and then tested for their ability to discriminate a reversal in the ordinal direction of the size changes. The method was modeled after Macchi Cassia et al. (2012, Experiment 2), who conducted a similar study with 4‐month‐old infants. To help newborns segregate the sequences during habituation, we presented them with three different stimulus sets, each featuring a unique form varying in size. To preclude newborns from using looming/zooming cues to discriminate between the familiar and the novel order at test, test stimuli increased/decreased in size by expanding/contracting horizontally, avoiding any approaching/retracting perception.

### 4.1 Methods

The methods were the same as in Experiment 2 except as follows.

#### 4.1.1 Participants

The final sample was composed of 44 healthy full‐term newborns (21 girls, mean age = 48 h, range = 20–101 h; mean birth weight = 3220 g; range = 2560–4040 g; APGAR score of 10 at 5 min). Data from 15 additional infants were discarded due to drowsiness or fussiness resulting in failure to complete all test trials (*N* = 10; 5 in the increasing and 5 in the decreasing habituation condition), experimenter error (*N* = 1) or looking times in at least one test trials shorter than 2 s (*N* = 4). Sample size estimation was based on Experiment 2. Participants were recruited following the same procedure as in Experiments 1 and 2, and tested only if in an alert state. They were randomly assigned to either the increasing or decreasing habituation condition, with no group differences in gestational age, birth weight, or age at testing (all *p*s > 0.13). Parents gave their written informed consent before testing began.

#### 4.1.2 Stimuli

**FIGURE 6:**
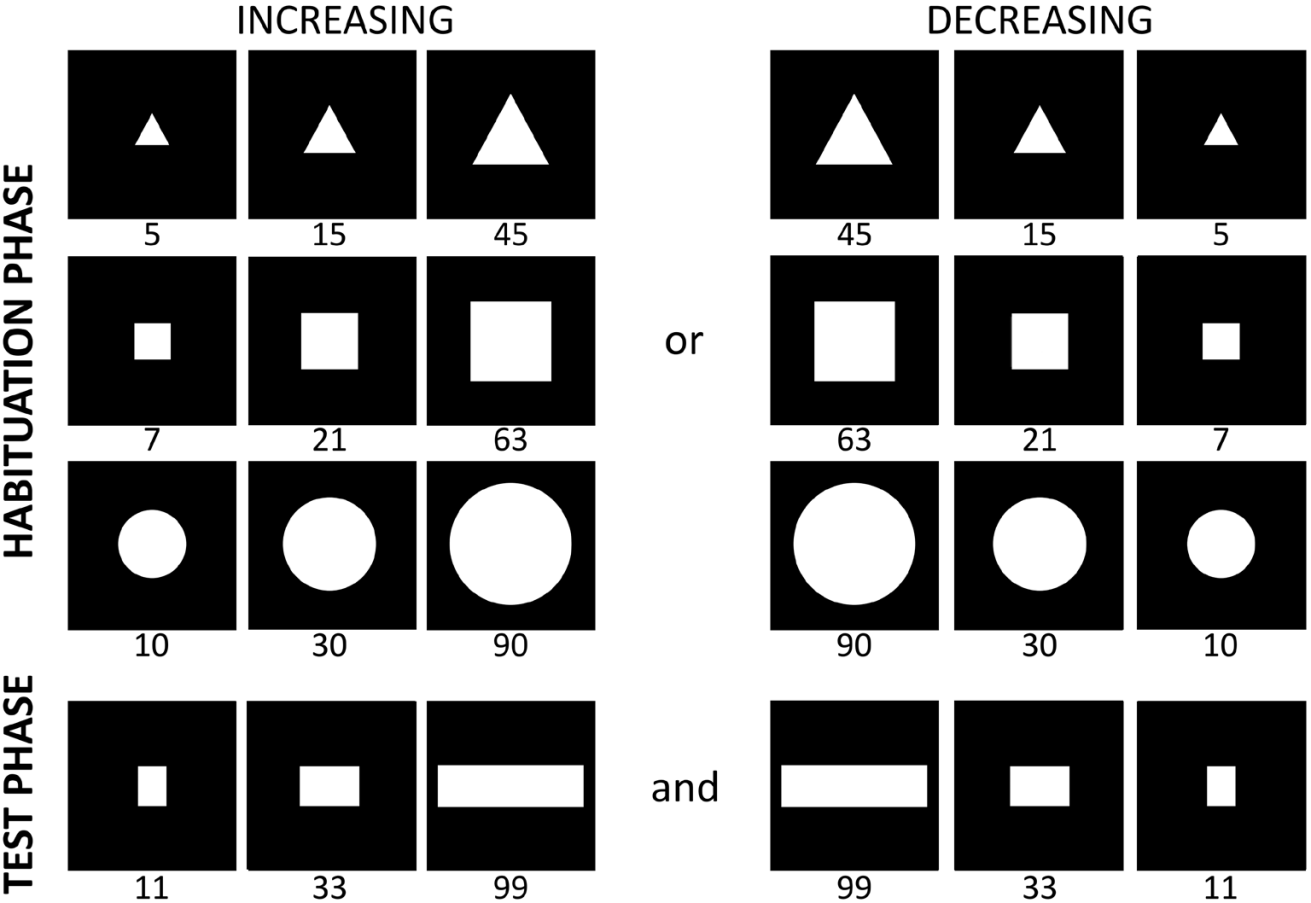
Stimuli in Experiment 3: Habituation and test sets. Panels on the left depict shapes presented to newborns in the increasing habituation condition, while panels on the right depict those in the decreasing condition. The top row shows the habituation sets (triangles, squares, circles) that expanded or contracted in both dimensions (vertically and horizontally). The bottom row shows the test sets (bars) that expanded or contracted horizontally only. Shape areas are reported in cm^2^. Shapes within each set maintained a constant area ratio of 1:3.

Stimuli were adapted from Macchi Cassia et al. (2012, Experiment 2). They were single white geometrical shapes that varied in area by a 1:3 ratio and were presented on a black background at the center of the screen. Like in Experiments 1 and 2, four sets of stimuli were used, each set featuring a different shape. The three sets used for the habituation phase contained triangles, squares, and circles that were, respectively, 5, 15, 45 cm^2^; 7, 21, 63 cm^2^; and 10, 30, 90 cm^2^. The test set contained three bars that were 11, 33, and 99 cm^2^ and expanded/contracted solely along the horizontal axis (Figure 6).

#### 4.1.3 Procedure

All aspects of the stimulus presentation procedure were identical to those used in Experiment 1 and 2 (Figure 2). Newborns were habituated to increasing or decreasing sequences of triangles, squares, and circles, and were then tested with both increasing and decreasing sequences containing newly sized bars (Figure 6). Like in Experiment 1 and 2, the three different habituation stimulus sets were presented in a consistent fixed order (either increasing or decreasing) across trials until newborns met the habituation criterion: from the smallest to the largest shape for the increasing condition (i.e., 5‐15‐45, 7‐21‐63, 10‐30‐90 cm^2^) and from the largest to the smallest square for the decreasing condition (i.e., 90‐30‐10, 63‐21‐7, 45‐15‐5 cm^2^).

For 27% of all trials, a third observer was involved in coding recorded data offline as the two initial coders' judgments differed by more than 5 s. Intercoder agreement between the two measurements, as assessed by Pearson's correlation on total fixation times on each of the four test trials, was *r* = 0.96. Statistical analyses were performed on log‐transformed averages of the two closest values for each trial.

### 4.2 Results and Discussion

**FIGURE 7:**
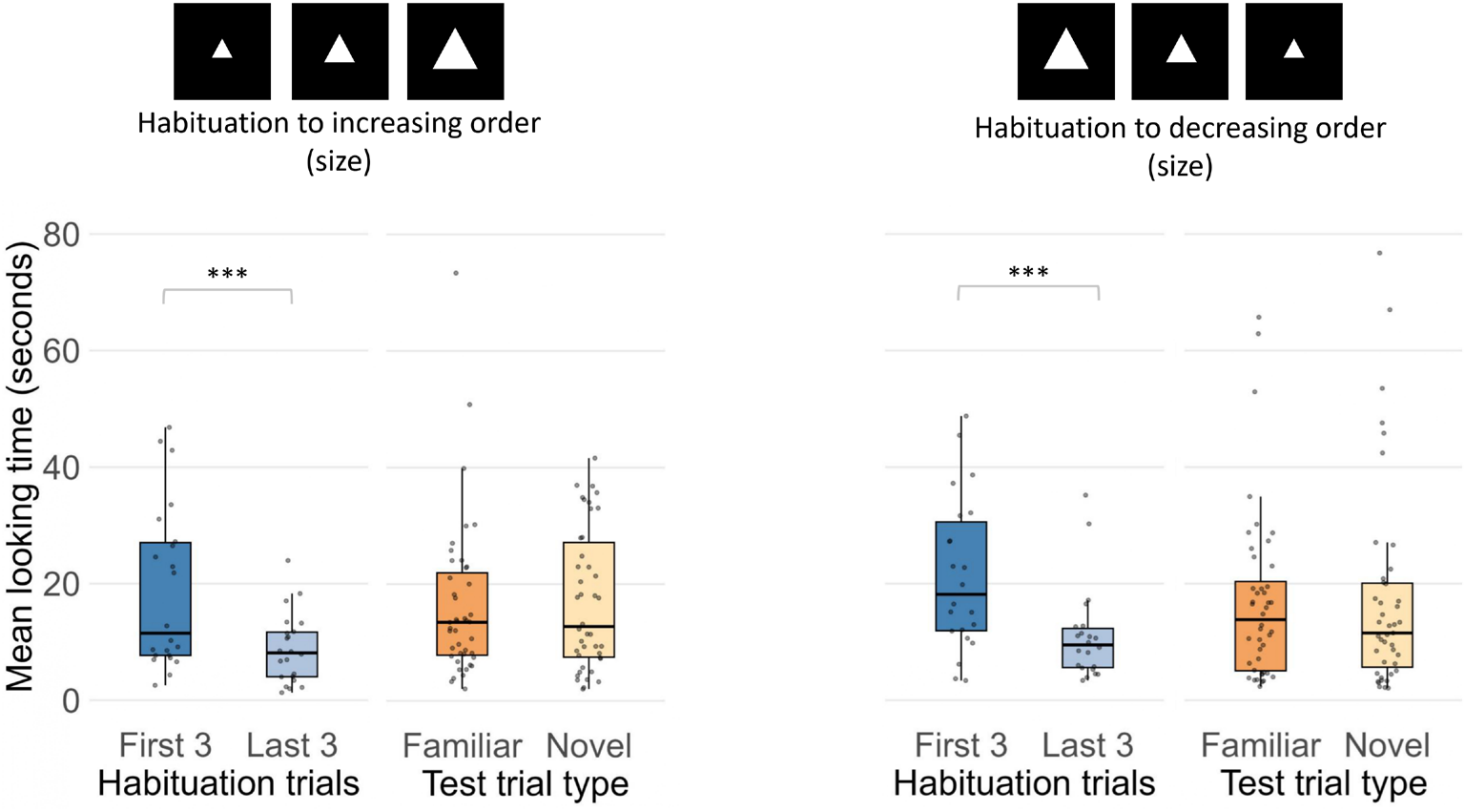
Mean total looking time in Experiment 3 to the first three and last three habituation trials and to familiar and novel test trials for newborns habituated to increasing (left) and decreasing (right) sequences. Black lines represent the median, the boxes indicate the interquartile range (IQR), dots show the individual data points. ***p < 0.001.

A repeated‐measure ANOVA on log‐transformed data with habituation order (increasing vs. decreasing) as a between‐subjects factor and habituation trials (first three vs. last three) as a within‐subjects factor confirmed the presence of a significant decrease in looking times between the first three (*M* = 20.13 s, SD = 2.05) and the last three habituation trials (*M* = 9.91 s, SD = 1.07), *F*(1,42) = 47.55, *p* < 0.001, *η* ^2^_p_ = 0.53, with no main effect or interaction involving habituation order (*p*s > 0.22) (Figure 7). Similar results were obtained from the ART analysis on raw looking times (*W*s < 0.87, *p*s < 0.007), which only revealed a main effect of habituation trials, *F*(1,42) = 39, *p* < 0.001, (all other *p*s > 0.35). No differences in log‐transformed (*t*(42) = 1.08; *p* = 0.9) and raw total habituation time (Wilcoxon = 203, *p* = 0.4; *W* < 9, *p* < 0.01), number of trials to habituate and proportional looking time decrement (Wilcoxon tests > 245, *p* > 0.6) were found across the two habituation order conditions: newborns required an average of 118.65 s (SD = 54.82) and 8.41 trials to habituate and showed a looking time decrement of 0.45 while viewing the increasing sequences, and required 146.42 s (SD = 93.44) and 7.73 trials to habituate to the decreasing sequences exhibiting a looking time decrement of 0.3.

To determine whether participants could distinguish the novel order from the familiar one during the test phase, a four‐way ANOVA was performed on log‐transformed looking times during test trials. The ANOVA, including habituation order (increasing vs. decreasing) and first test trial (familiar vs. novel) as between‐subjects factors and test trial pair (first vs. second) and test trial type (novel vs. familiar order) as within‐subjects factors, revealed a main effect of first test trial, *F*(1,40) = 5.67, *p* = 0.022, *η* ^2^_p_ = 0.12, with no other significant main effects or interactions, *p*s > 0.22 (Figure 7). Specifically, infants who first saw the familiar order at test (*M* = 22.6 s, SD = 14.54) showed overall longer looking times than those who were first presented with the novel order (*M* = 13.5 s, SD = 13.13). The ART analysis on raw looking times (*W*s < 0.87, *p*s < 0.007) also showed a significant main effect of first test trial, *F*(1, 40) = 6.89, *p* = 0.01, as well as a test trial pair main effect, *F*(1,120) = 5.75, *p* = 0.02, indicating that newborns' looking times increased during the second test trial pair (*M* = 96.9 s, SE = 6.48) compared to the first pair (*M* = 81.8 s, SE = 6.48). The number of infants who looked longer at the novel order did not differ from those who looked longer at the familiar one in either the increasing habituation condition (binomial test: 11 vs. 11, *p* = 0.58, one‐tailed) or the decreasing habituation condition (binomial test: 11 vs. 11, *p* = 0.58, one‐tailed).

#### 4.2.1 Comparison Across Experiment 2 and Experiment 3

Habituation performance across Experiment 2 and 3 was compared through a two‐way ANOVA on log‐transformed total habituation looking times and an ART analysis on raw total habituation time data including experiment (Experiment 2, Experiment 3) and habituation order (increasing, decreasing) as between‐subject factors. No significant effects were found. Nonetheless, the ART analysis (*W* = 0.8, *p* < 0.001) on proportional decline in looking times across the first three and the last three habituation trials revealed that the decrement was overall steeper in Experiment 2 (*M* = 0.51, SE = 3.7) compared to Experiment 3 (*M* = 0.38, SE = 3.7), *F*(1,84) = 6.05, *p* = 0.016, suggesting more efficient learning in the presence of multiple cues of ordinality.

To compare newborns' test performance across the two experiments, we performed a 2 (experiment) × 2 (habituation order) ANOVA on novelty preference scores (raw total novel looking times/raw total novel + total familiar). A significant habituation order main effect, *F*(1,84) = 6.29, *p* = 0.014, indicated that, across the two experiments, newborns habituated to increasing order (*M* = 51.9%, SE = 0.006) showed a larger novelty response compared to those habituated to the decreasing order (*M* = 49.5%, SE = 0.006). Despite that, one‐sample *t*‐tests comparing novelty preference scores to the chance level (50%) for each experiment and habituation condition confirmed that the novelty response was reliable only for newborns in the increasing habituation order condition of Experiment 2, *t*(21) = 4.4, *p* < 0.001 (all other *p*s > 0.52).

## 5 General Discussion

This study investigated the very developmental origins of numerical order processing, previously reported in infants as young as 4 months (de Hevia et al. 2017). We examined newborns' ability to represent and discriminate between visual sequences that increased and decreased in numerosity, in the absence (Experiment 1) and in the presence (Experiment 2) of additional non‐numerical quantitative cues to ordinality. Results showed that newborns only discriminated a reversal in order direction when the changes in numerosity were accompanied by corresponding changes in the continuous variables that typically accompany the increase/decrease in the number of elements in a scene, namely cumulative surface area and density. In Experiment 1, where ordinality was specified by variations in numerosity, newborns did not discriminate order reversal during the test. Newborns showed this ability only in Experiment 2 where, within the sequences, in addition to numerosity, the size of the individual items, the cumulative surface area occupied by them, and density also varied.

It is important to emphasize that our participants did not provide evidence of being able to discriminate a reversal in the direction of quantitative ordinal relations, either in the presence of numerical changes (Experiment 1) or in the absence of numerical changes (Experiment 3). Collectively, these findings suggest that newborns can compute ordinal operations on sequentially presented numerosities, comparing the approximate numerical content of perceptually available displays to that stored in working memory. However, this ability is contingent upon the integration of both numerical and non‐numerical cues, with neither type of information taking precedence. Newborns in Experiment 2 could successfully extract and represent ordinal relations based on concurrent variations in item numerosity, size, and cumulative area, but were unable to use any of these dimensions in isolation.

The finding that presenting multiple convergent quantitative cues facilitates infants to detect ordinal relations is not new in the literature. Suanda et al. (2008) reported similar evidence in 9‐month‐old infants, who failed to detect ordinal changes based on number, size, or cumulative area alone, but succeeded when all three cues were presented together. The authors discussed infants' failure with single cues as possibly due to their difficulty in focusing on the quantitative dimension that is diagnostic to ordinality while ignoring non‐monotonic changes in irrelevant dimensions. This may apply to the current study as well. In the habituation stimuli presented in Experiment 1, number increased, whereas items' size decreased, and cumulative area remained unchanged. In Experiment 3, size increased or decreased in the absence of numerical changes.

As an alternative, newborns' need for converging redundant quantitative cues may reflect the features of their early magnitude representation. There is evidence that representations of number, space, and time are interrelated at birth, as newborns can map changes in these fundamental dimensions when they vary in the same direction but not in opposite directions (de Hevia et al. 2014). It is therefore possible that the integration of converging and redundant information from multiple magnitude dimensions was necessary for newborns in the current study to achieve robust and reliable percepts that could then be compared to those previously and subsequently experienced to abstract the ordinal structure. Accordingly, newborns took longer to habituate to increasing sequences in Experiment 1 relying solely on numerosity and Experiment 3 relying only on non‐numerical quantitative cues compared to Experiment 2, where number covaried with item size and cumulative area. Similarly, the slope of looking times to habituate was steeper in Experiment 2 compared to both Experiments 1 and 3. This suggests that abstracting order based solely on numerical or size cues was more effortful and less efficient, while the presence of multiple cues of ordinality facilitated newborns in developing a robust representation of the ordinal relationships.

A second critical aspect of the findings of the reported studies is that newborns' ability to perform ordinal operations on visual sequences, where the number of items changes along with their physical size, is limited to increasing sequences, just as occurs in 4‐month‐old infants (de Hevia et al. 2017). Indeed, Experiment 2 showed faster habituation and a steeper decline in habituation looking times for increasing compared to decreasing sequences. This suggests that infants more readily formed a stable representation of the increasing order. Consequently, they also successfully discriminated the reversal in order direction at test in the increasing condition and not in the decreasing one. This provides the first evidence that the asymmetry signature in order processing, previously reported in older infants (de Hevia et al. 2017; Macchi Cassia et al. 2012), preschool‐aged children (Barth et al. 2008; Shinskey et al. 2009), adults (e.g., Ben‐Meir et al. 2012) of different cultures (Campbell and Xue 2001), and non‐human primates (Brannon and Terrace 1998; Cantlon and Brannon 2006; Judge et al. 2005), is present at the onset of postnatal life, before infants had any experience with quantitative changes deriving from transformations in the environment nor exposure to the growing dynamics of natural objects (Lakoff 2000).

In fact, the increasing order advantage could be interpreted within the framework of the Theory of Embodied Mathematics (Lakoff 2000) as emerging from our extensive experience with natural objects that grow in size across time. This may support the learning of associations between ‘early in time—small size’ and ‘later in time—large size’. This interpretation would fit with the finding that, while the increasing advantage has been observed in 4‐month‐old infants for both number (de Hevia et al. 2017) and size (Macchi Cassia et al. 2012), it is absent for the dimension of temporal duration (de Hevia et al. 2020), which is an abstract entity with no physical substrate. This suggests that the advantage may be grounded in our physical interactions with the world. Research on problem solving in adults offers insights into this topic by showing that individuals show a strong preference to add rather than subtract elements across a diverse range of simple everyday problems like creating symmetrical patterns, stabilizing artifacts, or editing text, showing what is called an ‘addition bias’ or ‘subtraction neglect’ to problem solving activities (Adams et al. 2021; Fischer et al. 2021). However, while postnatal experience with objects might contribute to this phenomenon, it cannot explain the presence of the increasing order advantage in 1‐ to‐4‐day‐old newborns.

It is therefore plausible that the advantage is rooted in biological constraints from our evolutionary history. One possibility is that such constraints include the alerting effect engendered by looming perception. It has been claimed that the attentional biases that occur when perceiving looming objects—objects that are perceived as approaching the observer—are examples of the phenomenon by which the perceptual systems have evolved to bestow specific advantages in survival and reproduction within a dynamic environment (Neuhoff 2018). Accordingly, there is ample evidence that looming objects are a very special class of stimuli that are treated with priority by both the auditory and visual systems in both humans (e.g., Franconeri and Simons 2003; McGuire et al. 2021) and non‐human animals (e.g., Ghazanfar et al. 2002; Maier et al. 2004). This same phenomenon is observed in newborns in both the visual (Orioli et al. 2018a, 2018b) and auditory (Ignatiadis et al. 2024) domains, confirming the potential of the evolutionary origin and universal basis of the bias.

The alerting effect associated with looming perception—i.e. the visual looming bias—could facilitate the processing of increasing ordinal relations embedded in looming stimuli. The current study, along with previous studies using single shapes (Macchi Cassia et al. 2012) and numerical displays (de Hevia et al. 2017), lacked objective looming effects due to stimulus design. Indeed, in the current studies, the appearance of each item within a sequence was separated by a 250‐ms blank screen. Furthermore, in Experiment 2, as the size of the items increased or decreased with numerosity, their spatial arrangement within the display also changed, thereby preventing the perception of a consistent looming effect. Nonetheless, increasing sequences and looming stimuli both entail increasing order. Given newborns' ability to associate representations of relative quantity across size and number (de Hevia et al. 2014), it is possible that they generalize the attentional response triggered by looming perception to all stimuli displaying increasing order, resulting in an early processing advantage for ordinal information in increasing sequences. While this advantage in visual habituation tasks may diminish over the first year, as 7‐month‐olds demonstrate equal ability to generalize increasing and decreasing order to novel numerosities at test (Picozzi et al. 2010), it may have cascading effects on arithmetic performance throughout the lifespan.

One aspect of the current findings may be at odds with this interpretation. While the asymmetry signature is present in 4‐month‐infants for both numerical (de Hevia et al. 2017) and size‐based sequences (Macchi Cassia et al. 2012), newborns in the current study failed to show evidence of representing order from increasing size. If the increasing advantage observed in Experiment 2 is rooted in the visual looming bias, why did such bias not generalize to size‐based sequences in Experiment 3? It is possible that this bias did generalize to size‐based sequences, even though newborns failed to recognize the familiar order in the test stimuli.

Null results in habituation studies can be challenging to interpret, and the lack of a novelty preference in the test phase does not necessarily indicate a lack of learning during habituation (see Colombo and Mitchell 2009; Roder et al. 2000). It is possible that infants did form a representation of the habituation stimulus, but the test stimuli did not sufficiently support generalization of the learned property. Indeed, while the habituation stimuli used in Experiment 3 varied in size by expanding/contracting along all axes, the test stimuli expanded/contracted solely along the horizontal axis. This was done precisely to avoid looming/zooming perception and prevent newborns from using such optical cues to generalize familiar order to the novel sizes at test. However, this could have introduced an element of novelty in both the familiar and novel stimuli that called newborns' attention to the novel shape, hindering recognition of the familiar order. This is a common phenomenon in infants' habituation studies in which the task requires recognition of a familiar stimulus dimension across strong perceptual modifications, where infants react to significant perceptual changes with an extensive re‐exploration of the familiar modified configuration to update their representation (e.g., Bulf and Turati 2010). Indeed, results of Experiment 3 revealed a significant Test trial order main effect, showing that looking times during test trials were overall longer for infants who first saw the familiar order than for those who first saw the novel order. This may suggest that the generalization of the order representation built during habituation to the new test sequences required an update of the representation itself to include the features of the new test stimuli, with the result that, once the representation was further enriched and strengthened, the dishabituation response was also more pronounced. Indeed, looking times of the infants who first saw the familiar order at test were at least 7 s longer than those of the infants who first saw the novel order in all single trials, both familiar and novel (all *p*s < 0.001, except the first novel trial, *p* = 0.064). Future studies could replicate Experiment 3 using more similar stimuli during habituation and test to investigate whether this factor might have influenced our results and determine whether, under such facilitating stimulus conditions, newborns represent size order.

An important aspect that needs to be acknowledged is the statistical power of our studies, particularly Experiments 1 and 3, which yielded null effects. Our sample sizes were determined a priori based on de Hevia et al. (2017), who reported a large effect size (Cohen's *f* = 0.53) for the habituation order × test trial type interaction in 4‐month‐old infants. This may have limited our ability to detect significant results in the more challenging conditions tested in Experiments 2 and 3, where novelty effects might have emerged with smaller effect sizes. To address this, we conducted a sensitivity analysis for each experiment, showing that, assuming a power of 0.95, the sample size for Experiment 3 was sufficient to detect a small‐to‐medium effect size (Cohen's *f* = 0.20), and Experiment 1 could detect a medium‐to‐large effect size (Cohen's *f* = 0.38) (input parameters and outputs available at https://osf.io/aezq6/?view_only=18aea219c7eb4c368f6ce9503e128bea). Of note, assuming a lower power of 0.80, the detectable effect size decreases to Cohen's *f* = 0.30 for Experiment 1 and to Cohen's *f* = 0.16 for Experiment 3. While future studies with larger sample sizes would be valuable to replicate these findings, we can nonetheless conclude that, under the same design parameters revealing asymmetrical processing of numerical order in 4‐month‐old infants (2017), the asymmetry signature is detectable at birth.

In conclusion, our findings demonstrate for the first time that newborns possess the ability to extract and represent ordinal relations between sequentially presented magnitudes. While this ability requires the integration of multiple quantitative dimensions, it provides a foundation for the development of more complex numerical and mathematical skills. The observed asymmetry in processing increasing and decreasing sequences, shared across animal species, suggests that the mechanisms underlying ordinal processing may have deep evolutionary roots, potentially linked to the perceptual biases associated with looming objects. Further research is needed to fully elucidate the specific factors influencing this asymmetry and its implications for the development of numerical and mathematical abilities.

## Data Availability

The manuscript has been screened using the StatCheck tool (http://statcheck.io) to verify the consistency between test statistics and reported *p*‐values. No inconsistencies were detected. The data and analytic code necessary to reproduce the analyses presented here are publicly accessible at the following URL: https://osf.io/aezq6/?view_only=18aea219c7eb4c368f6ce9503e128bea. The materials necessary to attempt to replicate the findings presented here are not publicly accessible, but available upon request to the first author. The analyses presented here were not preregistered.

## References

[cdev70025-bib-0001] Acerra, F. , Y. Burnod , and S. de Schonen . 2002. “Modelling Aspects of Face Processing in Early Infancy.” Developmental Science 5, no. 1: 98–117.

[cdev70025-bib-0002] Adachi, I. 2014. “Spontaneous Spatial Mapping of Learned Sequence in Chimpanzees: Evidence for a SNARC‐Like Effect.” PLoS One 9: 3. 10.1371/journal.pone.0090373 PMC3958337 24643044

[cdev70025-bib-0003] Adams, G. S. , B. A. Converse , A. H. Hales , and L. E. Klotz . 2021. “People Systematically Overlook Subtractive Changes.” Nature 592: 258–261. 33828317 10.1038/s41586-021-03380-y

[cdev70025-bib-0004] Atkinson, J. , O. J. Braddick , and J. French . 1979. “Contrast Sensitivity of the Human Neonate Measured by the Visual Evoked Potential.” Investigative Ophthalmology and Visual Science 18, no. 2: 210–212. 761974

[cdev70025-bib-0005] Aulet, L. S. , and S. F. Lourenco . 2021. “Numerosity and Cumulative Surface Area Are Perceived Holistically as Integral Dimensions.” Journal of Experimental Psychology: General 150, no. 1: 145. 32567881 10.1037/xge0000874 PMC7752852

[cdev70025-bib-0006] Bahrick, L. E. , and R. Lickliter . 2004. “Infants' Perception of Rhythm and Tempo in Unimodal and Multimodal Stimulation: A Developmental Test of the Intersensory Redundancy Hypothesis.” Cognitive, Affective, & Behavioral Neuroscience 4: 137–147. 10.3758/cabn.4.2.137 PMC2738586 15460921

[cdev70025-bib-0007] Ball, W. , and E. Tronick . 1971. “Infant Responses to Impending Collision: Optical and Real.” Science 171, no. 3973: 818–820. 5541165 10.1126/science.171.3973.818

[cdev70025-bib-0008] Banks, M. S. , and P. J. Bennett . 1991. “Anatomical and Physiological Constraints on Neonatal Visual Sensitivity and Determinants of Fixation Behavior.” In Newborn Attention: Biological Constraints and the Influence of Experience, edited by M. J. S. Weiss and P. R. Zelazo , 177–217. Ablex Publishing.

[cdev70025-bib-0009] Baroody, A. J. 1984. “Children's Difficulties in Subtraction: Some Causes and Questions.” Journal for Research in Mathematics Education 15, no. 3: 203–213.

[cdev70025-bib-0010] Barth, H. , L. Beckmann , and E. S. Spelke . 2008. “Nonsymbolic, Approximate Arithmetic in Children: Abstract Addition Prior to Instruction.” Developmental Psychology 44, no. 5: 1466. 18793077 10.1037/a0013046 PMC3489021

[cdev70025-bib-0011] Barth, H. , N. Kanwisher , and E. Spelke . 2003. “The Construction of Large Number Representations in Adults.” Cognition 86, no. 3: 201–221. 12485738 10.1016/s0010-0277(02)00178-6

[cdev70025-bib-0012] Barth, H. , K. La Mont , J. Lipton , S. Dehaene , N. Kanwisher , and E. Spelke . 2006. “Non‐Symbolic Arithmetic in Adults and Young Children.” Cognition 98, no. 3: 199–222. 15876429 10.1016/j.cognition.2004.09.011

[cdev70025-bib-0013] Ben‐Meir, S. , D. Ganor‐Stern , and J. Tzelgov . 2012. “Numerical and Physical Magnitudes Are Mapped Into Time.” Quarterly Journal of Experimental Psychology 65, no. 12: 2309–2320. 10.1080/17470218.2012.676656 22643130

[cdev70025-bib-0014] Blumberg, M. S. , and K. E. Adolph . 2023. “Protracted Development of Motor Cortex Constrains Rich Interpretations of Infant Cognition.” Trends in Cognitive Sciences 27, no. 3: 233–245. 36681607 10.1016/j.tics.2022.12.014 PMC9957955

[cdev70025-bib-0015] Bonn, C. D. , and J. F. Cantlon . 2017. “Spontaneous, Modality‐General Abstraction of a Ratio Scale.” Cognition 169: 36–45. 28806722 10.1016/j.cognition.2017.07.012 PMC5636217

[cdev70025-bib-0016] Bonn, C. D. , M. E. Netskou , A. Streri , and M. D. de Hevia . 2019. “The Association of Brightness With Number/Duration in Human Newborns.” PLoS One 14, no. 10: 1–23. 10.1371/journal.pone.0223192 PMC6773210 31574110

[cdev70025-bib-0017] Brannon, E. M. 2002. “The Development of Ordinal Numerical Knowledge in Infancy.” Cognition 83, no. 3: 223–240. 11934402 10.1016/s0010-0277(02)00005-7

[cdev70025-bib-0018] Brannon, E. M. , J. F. Cantlon , and H. S. Terrace . 2006a. “The Role of Reference Points in Ordinal Numerical Comparisons by Rhesus Macaques (*Macaca mulatta*).” Journal of Experimental Psychology: Animal Behavior Processes 32, no. 2: 120. 16634655 10.1037/0097-7403.32.2.120

[cdev70025-bib-0019] Brannon, E. M. , D. Lutz , and S. Cordes . 2006b. “The Development of Area Discrimination and Its Implications for Number Representation in Infancy.” Developmental Science 9, no. 6: F59–F64. 17059447 10.1111/j.1467-7687.2006.00530.x PMC1661837

[cdev70025-bib-0020] Brannon, E. M. , and H. S. Terrace . 1998. “Ordering of the Numerosities 1 to 9 by Monkeys.” Science 282, no. 5389: 746–749. 9784133 10.1126/science.282.5389.746

[cdev70025-bib-0021] Brown, A. M. , F. O. Opoku , and M. R. Stenger . 2018. “Neonatal Contrast Sensitivity and Visual Acuity: Basic Psychophysics.” Translational Vision Science & Technology 7, no. 3: 18. 10.1167/tvst.7.3.18 PMC6016435 29946492

[cdev70025-bib-0022] Brown, A. M. , and M. Yamamoto . 1986. “Visual Acuity in Newborn and Preterm Infants Measured With Grating Acuity Cards.” American Journal of Ophthalmology 102, no. 2: 245–253. 3740187 10.1016/0002-9394(86)90153-4

[cdev70025-bib-0023] Bulf, H. , E. Quadrelli , S. Brady , B. Nguyen , V. Macchi Cassia , and S. P. Johnson . 2021. “Rule Learning Transfer Across Linguistic and Visual Modalities in 7‐Month‐Old Infants.” Infancy 26, no. 3: 442–454. 33709450 10.1111/infa.12397

[cdev70025-bib-0024] Bulf, H. , and C. Turati . 2010. “The Role of Rigid Motion in Newborns' Face Recognition.” Visual Cognition 18, no. 4: 504–512.

[cdev70025-bib-0025] Campbell, J. I. D. , and Q. Xue . 2001. “Cognitive Arithmetic Across Cultures.” Journal of Experimental Psychology: General 130, no. 2: 299. 11409105 10.1037//0096-3445.130.2.299

[cdev70025-bib-0026] Canobi, K. H. 2005. “Children's Profiles of Addition and Subtraction Understanding.” Journal of Experimental Child Psychology 92, no. 3: 220–246. 16024038 10.1016/j.jecp.2005.06.001

[cdev70025-bib-0027] Cantlon, J. F. 2012. “Math, Monkeys, and the Developing Brain.” Proceedings of the National Academy of Sciences of the United States of America 109, no. Suppl.1: 10725–10732. 22723349 10.1073/pnas.1201893109 PMC3386867

[cdev70025-bib-0028] Cantlon, J. F. , and E. M. Brannon . 2006. “Shared System for Ordering Small and Large Numbers in Monkeys and Humans.” Psychological Science 17, no. 5: 401–406. 16683927 10.1111/j.1467-9280.2006.01719.x

[cdev70025-bib-0029] Carey, S. 2011. “Précis of the Origin of Concepts.” Behavioral and Brain Sciences 34, no. 3: 113–124. 21676291 10.1017/S0140525X10000919 PMC3489495

[cdev70025-bib-0030] Cléry, J. , O. Guipponi , S. Odouard , C. Wardak , and S. B. Hamed . 2015. “Impact Prediction by Looming Visual Stimuli Enhances Tactile Detection.” Journal of Neuroscience 35, no. 10: 4179–4189. 25762665 10.1523/JNEUROSCI.3031-14.2015 PMC6605290

[cdev70025-bib-0031] Colombo, J. , and D. W. Mitchell . 2009. “Infant Visual Habituation.” Neurobiology of Learning and Memory 92, no. 2: 225–234. 18620070 10.1016/j.nlm.2008.06.002 PMC2758574

[cdev70025-bib-0032] Cooper, R. G., Jr. 1984. “Early Number Development: Discovering number space with addition and subtraction.” In The 18th Annual Carnegie Symposium on Cognition: Origins of cognitive skills, C. Sophian (Ed.), 157–192.

[cdev70025-bib-0033] Cordes, S. , and E. M. Brannon . 2008. “Quantitative Competencies in Infancy.” Developmental Science 11, no. 6: 803–808. 19046148 10.1111/j.1467-7687.2008.00770.x

[cdev70025-bib-0034] Cordes, S. , and E. M. Brannon . 2011. “Attending to One of Many: When Infants Are Surprisingly Poor at Discriminating an Item's Size.” Frontiers in Psychology 2: 65. 21687440 10.3389/fpsyg.2011.00065 PMC3110486

[cdev70025-bib-0035] Coubart, A. , V. Izard , E. S. Spelke , J. Marie , and A. Streri . 2014. “Dissociation Between Small and Large Numerosities in Newborn Infants.” Developmental Science 17, no. 1: 11–22. 10.1111/desc.12108. 24267592 PMC4624260

[cdev70025-bib-0036] Csibra, G. , M. Hernik , O. Mascaro , D. Tatone , and M. Lengyel . 2016. “Statistical Treatment of Looking‐Time Data.” Developmental Psychology 52, no. 4: 521. 26845505 10.1037/dev0000083 PMC4817233

[cdev70025-bib-0037] de Hevia, M. D. 2016. “Core Mathematical Abilities in Infants: Number and Much More.” Progress in Brain Research 227: 53–74. 27339008 10.1016/bs.pbr.2016.04.014

[cdev70025-bib-0038] de Hevia, M. D. , M. Addabbo , E. Nava , E. Croci , L. Girelli , and V. Macchi Cassia . 2017. “Infants' Detection of Increasing Numerical Order Comes Before Detection of Decreasing Number.” Cognition 158: 177–188. 10.1016/j.cognition.2016.10.022. 27835788

[cdev70025-bib-0039] de Hevia, M. D. , V. Izard , A. Coubart , E. S. Spelke , and A. Streri . 2014. “Representations of Space, Time, and Number n Neonates.” Proceedings of the National Academy of Sciences of the United States of America 111, no. 13: 4809–4813. 10.1073/PNAS.1323628111. 24639511 PMC3977279

[cdev70025-bib-0040] de Hevia, M. D. , V. Macchi Cassia , L. Veggiotti , and M. E. Netskou . 2020. “Discrimination of Ordinal Relationships in Temporal Sequences by 4‐Month‐Old Infants.” Cognition 195: 104091. 10.1016/j.cognition.2019.104091. 31739006

[cdev70025-bib-0041] de Hevia, M. D. , and E. S. Spelke . 2010. “Number‐Space Mapping in Human Infants.” Psychological Science 21, no. 5: 653–660. 10.1177/0956797610366091. 20483843 PMC3129621

[cdev70025-bib-0042] de Hevia, M. D. , L. Veggiotti , and Y. Baqqali . 2025. “Spatial Associations of Number and Pitch in Human Newborns.” Journal of Experimental Child Psychology 256: 106259. 40273466 10.1016/j.jecp.2025.106259

[cdev70025-bib-0043] Dehaene, S. 2011. The Number Sense: How the Mind Creates Mathematics. Oxford University Press.

[cdev70025-bib-0044] Drucker, C. B. , and E. M. Brannon . 2014. “Rhesus Monkeys (*Macaca mulatta*) Map Number Onto Space.” Cognition 132, no. 1: 57–67. 24762923 10.1016/j.cognition.2014.03.011 PMC4031030

[cdev70025-bib-0045] Dunn, H. , N. Bernstein , M. D. de Hevia , V. M. Cassia , H. Bulf , and K. McCrink . 2019. “Operational Momentum for Magnitude Ordering in Preschool Children and Adults.” Journal of Experimental Child Psychology 179: 260–275. 30562633 10.1016/j.jecp.2018.11.017 PMC6311425

[cdev70025-bib-0046] Feigenson, L. 2011. “Predicting Sights From Sounds: 6‐Month‐Olds' Intermodal Numerical Abilities.” Journal of Experimental Child Psychology 110, no. 3: 347–361. 21616502 10.1016/j.jecp.2011.04.004 PMC3139716

[cdev70025-bib-0047] Fischer, M. H. , B. Winter , A. Felisatti , A. Myachykov , M. A. Mende , and S. Shaki . 2021. “More Instructions Make Fewer Subtractions.” Frontiers in Psychology 12: 720616. 34650481 10.3389/fpsyg.2021.720616 PMC8506214

[cdev70025-bib-0048] Franconeri, S. L. , and D. J. Simons . 2003. “Moving and Looming Stimuli Capture Attention.” Perception & Psychophysics 65, no. 7: 999–1010. 14674628 10.3758/bf03194829

[cdev70025-bib-0049] Fuson, K. 1984. “More Complexities in Subtraction.” Journal for Research in Mathematics Education 15, no. 3: 214–225.

[cdev70025-bib-0050] Garland, A. , and J. Low . 2014. “Addition and Subtraction in Wild New Zealand Robins.” Behavioural Processes 109: 103–110. 25193352 10.1016/j.beproc.2014.08.022

[cdev70025-bib-0051] Gatto, E. , O. J. Loukola , and C. Agrillo . 2022. “Quantitative Abilities of Invertebrates: A Methodological Review.” Animal Cognition 25, no. 1: 5–19. 34282520 10.1007/s10071-021-01529-w PMC8904327

[cdev70025-bib-0052] Geers, L. , P. Kozieja , and Y. Coello . 2024. “Multisensory Peripersonal Space: Visual Looming Stimuli Induce Stronger Response Facilitation to Tactile Than Auditory and Visual Stimulations.” Cortex 173: 222–233. 38430652 10.1016/j.cortex.2024.01.008

[cdev70025-bib-0053] Ghazanfar, A. A. , J. G. Neuhoff , and N. K. Logothetis . 2002. “Auditory Looming Perception in Rhesus Monkeys.” Proceedings of the National Academy of Sciences 99, no. 24: 15755–15757. 10.1073/pnas.242469699 PMC137788 12429855

[cdev70025-bib-0054] Ignatiadis, K. , D. Baier , R. Barumerli , I. Sziller , B. Tóth , and R. Baumgartner . 2024. “Cortical Signatures of Auditory Looming Bias Show Cue‐Specific Adaptation Between Newborns and Young Adults.” Communications Psychology 2, no. 1: 56. 38859821 10.1038/s44271-024-00105-5 PMC11163589

[cdev70025-bib-0055] Izard, V. R. , C. Sann , E. S. Spelke , and A. Streri . 2009. “Newborn Infants Perceive Abstract Numbers.” Proceedings of the National Academy of Sciences of the United States of America 106, no. 25: 10382–10385. 10.1073/PNAS.0812142106. 19520833 PMC2700913

[cdev70025-bib-0056] Jordan, K. E. , S. H. Suanda , and E. M. Brannon . 2008. “Intersensory Redundancy Accelerates Preverbal Numerical Competence.” Cognition 108, no. 1: 210–221. 18226807 10.1016/j.cognition.2007.12.001 PMC2768652

[cdev70025-bib-0057] Judge, P. G. , T. A. Evans , and D. K. Vyas . 2005. “Ordinal Representation of Numeric Quantities by Brown Capuchin Monkeys (*Cebus apella*).” Journal of Experimental Psychology: Animal Behavior Processes 31, no. 1: 79. 15656729 10.1037/0097-7403.31.1.79

[cdev70025-bib-0058] Kamii, C. , B. A. Lewis , and L. D. Kirkland . 2001. “Fluency in Subtraction Compared With Addition.” Journal of Mathematical Behavior 20, no. 1: 33–42.

[cdev70025-bib-0059] Kandula, M. , D. Hofman , and H. C. Dijkerman . 2015. “Visuo‐Tactile Interactions Are Dependent on the Predictive Value of the Visual Stimulus.” Neuropsychologia 70: 358–366. 25498404 10.1016/j.neuropsychologia.2014.12.008

[cdev70025-bib-0060] Kirkham, N. Z. , J. A. Slemmer , D. C. Richardson , and S. P. Johnson . 2007. “Location, Location, Location: Development of Spatiotemporal Sequence Learning in Infancy.” Child Development 78, no. 5: 1559–1571. 17883448 10.1111/j.1467-8624.2007.01083.x

[cdev70025-bib-0061] Kobayashi, T. , K. Hiraki , R. Mugitani , and T. Hasegawa . 2004. “Baby Arithmetic: One Object Plus One Tone.” Cognition 91, no. 2: B23–B34. 10.1016/j.cognition.2003.09.004. 14738775

[cdev70025-bib-0062] Lakoff, G. 2000. Where Mathematics Comes From. Basic Books.

[cdev70025-bib-0063] Libertus, M. E. , A. Starr , and E. M. Brannon . 2014. “Number Trumps Area for 7‐Month‐Old Infants.” Developmental Psychology 50, no. 1: 108. 23647413 10.1037/a0032986 PMC3796133

[cdev70025-bib-0064] Livingstone, M. S. , W. W. Pettine , K. Srihasam , B. Moore , I. A. Morocz , and D. Lee . 2014. “Symbol Addition by Monkeys Provides Evidence for Normalized Quantity Coding.” Proceedings of the National Academy of Sciences of the United States of America 111, no. 18: 6822–6827. 10.1073/pnas.1404208111. 24753600 PMC4020100

[cdev70025-bib-0066] Macchi Cassia, V. , H. Bulf , K. McCrink , and M. D. De Hevia . 2017. “Operational Momentum During Ordering Operations for Size and Number in 4‐Month‐Old Infants.” Journal of Numerical Cognition 3, no. 2: 270–287. 10.5964/jnc.v3i2.67.

[cdev70025-bib-0067] Macchi Cassia, V. , K. McCrink , M. D. Hevia , V. Gariboldi , and H. Bulf . 2016. “Operational Momentum and Size Ordering in Preverbal Infants.” Psychological Research 80, no. 3: 360–367. 26898647 10.1007/s00426-016-0750-9 PMC5319925

[cdev70025-bib-0065] Macchi Cassia, V. , M. Picozzi , L. Girelli , and M. D. de Hevia . 2012. “Increasing Magnitude Counts More: Asymmetrical Processing of Ordinality in 4‐Month‐Old Infants.” Cognition 124, no. 2: 183–193. 10.1016/j.cognition.2012.05.004. 22676954

[cdev70025-bib-0068] Maier, J. X. , J. G. Neuhoff , N. K. Logothetis , and A. A. Ghazanfar . 2004. “Multisensory Integration of Looming Signals by Rhesus Monkeys.” Neuron 43, no. 2: 177–181. 15260954 10.1016/j.neuron.2004.06.027

[cdev70025-bib-0069] McCrink, K. , and K. Wynn . 2004. “Large‐Number Addition and Subtraction by 9‐Month‐Old Infants.” Psychological Science 15, no. 11: 776–781. 10.1111/j.0956-7976.2004.00755.x. 15482450

[cdev70025-bib-0070] McCrink, K. , and K. Wynn . 2009. “Operational Momentum in Large‐Number Addition and Subtraction by 9‐Month‐Olds.” Journal of Experimental Child Psychology 103, no. 4: 400–408. 19285683 10.1016/j.jecp.2009.01.013

[cdev70025-bib-0071] McGuire, A. , A. Ciersdorff , O. Gillath , and M. Vitevitch . 2021. “Effects of Cognitive Load and Type of Object on the Visual Looming Bias.” Attention, Perception, & Psychophysics 83: 1508–1517. 10.3758/s13414-021-02271-8 33751451

[cdev70025-bib-0072] Mix, K. S. , J. Huttenlocher , and S. C. Levine . 2002. Quantitative Development in Infancy and Early Childhood. Oxford University Press.

[cdev70025-bib-0073] Müller, D. , and W. Schwarz . 2008. ““1‐2‐3”: Is There a Temporal Number Line? Evidence From a Serial Comparison Task.” Experimental Psychology 55, no. 3: 143–150. 18549160 10.1027/1618-3169.55.3.143

[cdev70025-bib-0074] Náñez, J., Sr. 1988. “Perception of Impending Collision in 3‐To 6‐Week‐Old Human Infants.” Infant Behavior and Development 11, no. 4: 447–463.

[cdev70025-bib-0075] Neuhoff, J. G. 2018. “Adaptive Biases in Visual and Auditory Looming Perception.” In Spatial biases in perception and cognition, T. L. Hubbard (Ed.), 180–190. Cambridge University Press.

[cdev70025-bib-0076] Orioli, G. , A. J. Bremner , and T. Farroni . 2018a. “Multisensory Perception of Looming and Receding Objects in Human Newborns.” Current Biology 28, no. 22: R1294–R1295. 30458145 10.1016/j.cub.2018.10.004

[cdev70025-bib-0077] Orioli, G. , M. L. Filippetti , W. Gerbino , D. Dragovic , and T. Farroni . 2018b. “Trajectory Discrimination and Peripersonal Space Perception in Newborns.” Infancy 23, no. 2: 252–267. 29541001 10.1111/infa.12207 PMC5836937

[cdev70025-bib-0078] Pascalis, O. , and M. de Haan . 2014. “Recognition Memory and Novelty Preference: What Model?” In Progress in Infancy Research, 95–119. Psychology Press.

[cdev70025-bib-0079] Perry, L. K. , L. K. Samuelson , L. M. Malloy , and R. N. Schiffer . 2010. “Learn Locally, Think Globally: Exemplar Variability Supports Higher‐Order Generalization and Word Learning.” Psychological Science 21, no. 12: 1894–1902. 21106892 10.1177/0956797610389189 PMC3144952

[cdev70025-bib-0080] Piaget, J. 1954. “The Child's Conception of Number.” Journal of Consulting Psychology 18, no. 1: 76.

[cdev70025-bib-0081] Picozzi, M. , M. D. de Hevia , L. Girelli , and V. Macchi Cassia . 2010. “Seven‐Month‐Olds Detect Ordinal Numerical Relationships Within Temporal Sequences.” Journal of Experimental Child Psychology 107, no. 3: 359–367. 10.1016/j.jecp.2010.05.005. 20579661

[cdev70025-bib-0082] Roder, B. J. , E. W. Bushnell , and A. M. Sasseville . 2000. “Infants' Preferences for Familiarity and Novelty During the Course of Visual Processing.” Infancy 1, no. 4: 491–507. 32680296 10.1207/S15327078IN0104_9

[cdev70025-bib-0083] Rugani, R. , M. Loconsole , F. Simion , and L. Regolin . 2020. “Individually Distinctive Features Facilitate Numerical Discrimination of Sets of Objects in Domestic Chicks.” Scientific Reports 10, no. 1: 1. 33009471 10.1038/s41598-020-73431-3 PMC7532216

[cdev70025-bib-0084] Rugani, R. , G. Vallortigara , K. Priftis , and L. Regolin . 2015. “Number‐Space Mapping in the Newborn Chick Resembles Humans' Mental Number Line.” Science 347, no. 6221: 534–536. 25635096 10.1126/science.aaa1379

[cdev70025-bib-0085] Shinskey, J. L. , C. H. Chan , R. Coleman , L. Moxom , and E. Yamamoto . 2009. “Preschoolers' Nonsymbolic Arithmetic With Large Sets: Is Addition More Accurate Than Subtraction?” Journal of Experimental Child Psychology 103, no. 4: 409–420. 19285685 10.1016/j.jecp.2009.01.012

[cdev70025-bib-0086] Simion, F. , L. Regolin , and H. Bulf . 2008. “A Predisposition for Biological Motion in the Newborn Baby.” Proceedings of the National Academy of Sciences of the United States of America 105, no. 2: 809–813. 18174333 10.1073/pnas.0707021105 PMC2206618

[cdev70025-bib-0087] Suanda, S. H. , W. Tompson , and E. M. Brannon . 2008. “Changes in the Ability to Detect Ordinal Numerical Relationships Between 9 and 11 Months of Age.” Infancy 13, no. 4: 308–337. 10.1080/15250000802188800. 20703362 PMC2918911

[cdev70025-bib-0088] Van der Weel, F. R. , and A. L. H. van der Meer . 2009. “Seeing It Coming: Infants' Brain Responses to Looming Danger.” Naturwissenschaften 96: 1385–1391. 19756463 10.1007/s00114-009-0585-y

[cdev70025-bib-0089] Wynn, K. 1992. “Addition and Subtraction by Human Infants.” Nature 358, no. 6389: 749–750. 1508269 10.1038/358749a0

[cdev70025-bib-0090] Xu, F. , and E. S. Spelke . 2000. “Large Number Discrimination in 6‐Month‐Old Infants.” Cognition 74, no. 1: 1–11. 10.1016/S0010-0277(99)00066-9. 10594312

